# Fluorescent gold nanoclusters possess multiple actions against atherosclerosis

**DOI:** 10.1016/j.redox.2024.103427

**Published:** 2024-11-12

**Authors:** Yi-Nan Lee, Yih-Jer Wu, Cheng-Huang Su, Bo-Jeng Wang, Sheng-Hsun Yang, Hsin-I Lee, Yen-Hung Chou, Ting-Yi Tien, Chao-Feng Lin, Wen-Hsiung Chan, Ching-Hu Chung, Shin-Wei Wang, Hung-I Yeh

**Affiliations:** a Cardiovascular Center, Department of Internal Medicine, and Department of Medical Research, MacKay Memorial Hospital, Taipei, 10449, Taiwan; b Department of Medicine, MacKay Medical College, New Taipei City, 25245, Taiwan; c Department of Bioscience Technology and Center for Nanotechnology, Chung Yuan Christian University, Zhongbei Road, Zhongli District, Taoyuan City, 32023, Taiwan; d Institute of Biomedical Sciences, MacKay Medical College, New Taipei City, 25245, Taiwan

**Keywords:** Cholesterol-lowering, Anti-inflammation, Plaque burden, KEAP1, Nanomole

## Abstract

Atherosclerosis caused major morbidity and mortality worldwide. Molecules possessing lipid-lowering and/or anti-inflammatory properties are potential druggable targets against atherosclerosis. We examined the anti-atherosclerotic effects of fluorescent gold nanoclusters (FANC), which were dihydrolipoic acid (DHLA)-capped 2-nm gold nanoparticles. We evaluated the 8-week effects of FANC in Western-type diet-fed *ApoE*-deficient mice by either continuous intraperitoneal delivery **(**20 μM, 50 μl weekly**)** or via drinking water (300 nM). FANC reduced aortic atheroma burden, serum total cholesterol, and oxidative stress markers malondialdehyde and 4-hydroxynonenal levels. FANC attenuated hepatic lipid deposit, with changed expression of lipid homeostasis-related genes HMGCR, SREBP, PCSK9, and LDLR in a pattern similar to mice treated with ezetimibe. FANC also inhibited intestinal cholesterol absorption, resembling the action of ezetimibe. The lipid-lowering and anti-atherosclerotic effects of FANC reappeared in Western-type diet-fed *LDLr*-deficient mice. FANC bound insulin receptor β (IRβ) via DHLA, leading to AKT activation. However, unlike insulin, which also bound IRβ to activate AKT to induce HO-1, activation of AKT by FANC was independent of HO-1 expression in human aortic endothelial cells (HAECs). Alternatively, FANC up-regulated NRF2, interfered the binding of KEAP1 to NRF2, and promoted KEAP1 degradation to free NRF2 for nuclear entry to induce HO-1 that suppressed the expression of ICAM-1 and VCAM-1. Consistently, FANC suppressed ox-LDL-induced enhanced attachment of THP-derived macrophages onto HAECs. In macrophages, FANC up-regulated ABCA1, and reversed ox-LDL-induced suppression of cholesterol efflux. FANC effected *in vitro* at nano moles. In conclusion, our findings showed novel actions and multiple mechanisms of FANC worked coherently against atherosclerosis.

## Abbreviations

4-HNE: 4-hydroxynonenal
ABCA1: ATP-binding cassette transporter A1
AKT: protein kinase B (PKB)
HAECs: Human Aortic Endothelial Cells
ApoE: apolipoprotein E
ASCVD: Atherosclerotic cardiovascular diseases
AuNPs: gold nanoparticles
DHLA: dihydrolipoic acid
EDC: N-Ethyl-N’-(3-dimethylaminopropyl) carbodiimide
Ez: ezetimibe
FANC: fluorescent gold nanoclusters
HDL-C: high-density lipoprotein cholesterol
HMGCR: 3-hydroxy-3-methylglutaryl-CoA reductase
HO-1: heme oxygenase 1
ICAM-1: intercellular adhesion molecule 1
IL-6: interleukin-6
IRβ: insulin receptor beta
IP: Intraperitoneal injection
KEAP1: Kelch-like ECH (KEAP1)-associated protein 1
LDL-C: low density lipoprotein-cholesterol
LDLR: low density lipoprotein receptor
LPS: lipopolysaccharide
ox-LDL: oxidized-low density lipoprotein
MDA: malondialdehyde
NQO1: NAD(P)H: quinone oxidoreductase 1
NPC1L1: Niemann-Pick C1-Like 1
NRF2: NF-E2-related factor 2
PCSK9: proprotein convertase subtilisin/kexin type 9
PMA: phorbol 12-myristate 13-acetate
SREBP-1: Sterol Regulatory Element-binding Protein-1
TNF-α: tumor necrosis factor-alpha
VCAM-1: vascular cell adhesion molecule 1

## 1 Introduction

Atherosclerotic cardiovascular diseases (ASCVD), including coronary artery disease, stroke, and peripheral artery disease, are the leading cause of morbidity and mortality worldwide [1]. The pathogenesis of atherosclerosis is multi-factorial, and cholesterol and inflammation play key roles to affect the homeostasis of endothelial cells [[2], [3], [4]]. Epidemiologic studies have shown that blood cholesterol levels, in particular, low density lipoprotein-cholesterol (LDL-C), are causatively linked to ASCVD [5]. Clinical trials have shown that lipid-lowering strategies to reduce blood level of LDL-C, such as diet control, surgical resection of intestine, and pharmacological intervention reduced the formation of atheroma burden and improved clinical outcomes of ASCVD [6].

The pathological findings of arterial wall in people with ASCVD are compatible with chronic inflammation, in which monocytes/macrophages play a key role. At initial stage of atherosclerosis, expression of adhesion molecules, such as intercellular adhesion molecule 1 (ICAM-1) and vascular cell adhesion molecule 1 (VCAM-1) [7,8], by the vascular endothelial cells attract circulating monocytes to attach the luminal side of artery and penetrate the intimal layer, with subsequent uptake of cholesterol in the vascular wall to become foam cells and accumulate to form plaques. Anti-inflammatory agents have been tested in patients with ASCVD with promising results. For example, canakizumab, a monoclonal antibody against interleukin 1-beta (IL-1β), has been shown effectively improved the clinical outcomes of ASCVD [9]. Therefore, molecules possessing lipid-lowering and anti-inflammatory properties are potential druggable targets against ASCVD.

We have reported that FANC made of 2-nm gold nanoparticles (AuNPs) and capped with DHLA, the reduced form of α-lipoic acid, give high colloidal stability in aqueous solution for months, are potent anti-oxidants and exert anti-inflammation action [12,13]. Accumulated studies have shown that each of the components of FANC, AuNPs and α-lipoic acid, possessed antioxidant and anti-inflammation activity [10,11]. AuNPs have been reported to enhance the activity of other anti-oxidants [[14], [15], [16]]. For example, the antioxidant activity of vitamin E analogue had been increased eight times by assembly on AuNPs [12]. Hypercholesterolemia is associated with increased oxidative stress and induces inflammation [17,18]. Endogenous antioxidant capacity is increased under excess reactive oxygen species (ROS) exposure to induce the Kelch-like ECH associated protein 1 (KEAP1)-NF-E2-related factor 2 (NRF2)-mediated antioxidant gene expression, including heme oxygenase 1 (HO-1) and NAD(P)H: quinone oxidoreductase 1 (NQO1) [19], which are also involved in anti-inflammation. In fact, this NRF2-KEAP1 signaling axis is involved in the development and progression of several cancerous and non-cancerous diseases [[20], [21], [22]]. Medical application of AuNPs is an emerging field of translational research in drug delivery system, photothermal therapy and as a therapeutic agent [[23], [24], [25]]. Up to date, clinical trials with therapeutic AuNPs were agents for amyotrophic lateral sclerosis, relapsing multiple sclerosis and Parkinson's disease in Phase I and Phase II [[9], [26], [27], [28]]. However, to our best knowledge, there is no report of clinical application of AuNPs, including FANC, in ASCVD.

In this study, to determine FANC against atherosclerosis, we evaluated the therapeutic effects of FANC on reducing atherosclerotic plaque burden using apolipoprotein E (*ApoE*)-deficient mice and low density lipoprotein receptor (*LDLR*)-deficient mice fed high fat diet. In addition, we explored the underlying mechanisms by examining cholesterol regulation, pro-inflammatory and antioxidant status of endothelial cells and cholesterol efflux of macrophages in response to FANC, the interaction between cells and between molecules, and involved signaling pathways. These findings could contribute to the understanding of the clinical potential of FANC in ASCVD.

## 2 Materials and methods

### 2.1 Animals and ethical statements

The Institutional Animal Care and Use Committee of MacKay Memorial Hospital had approved our research protocol (MMH-A-S-109-24), according to the Guide for the Care and Use of Laboratory Animals, published by the US National Institutes of Health (NIH publication, 8th Edition, 2011). All animal experiments were performed in accordance with the guidelines of the committee, with efforts made to minimize the suffering of the animals. *ApoE*-deficient mice on a C57BL/6J background and wild-type littermate male mice were purchased from National Laboratory Animal Center (NLAC, Taipei) for the experiments. The mice were bred and housed under pathogen-free conditions in isolated cages on a 12-h light/dark cycle, and left freely access to standard chow, Western-type diet (containing 0.21 (gm%) cholesterol, 21 (gm%) fat, 20 (gm%) protein, 50 (gm%) carbohydrate, #D12079B, Research Diets, New Brunswick, NJ, USA). Ezetimibe was given using costumed Western-type diet (0.005%, w/w).

### 2.2 Osmotic minipump implantation

Eight week-old animals were anesthetized with intraperitoneal injection of pentobarbital (80 mg/kg). Osmotic minipumps (Model 2004, Alzet, CA, USA), filled with 200 μl of either FANCs (20 μM) or PBS (control), were implanted intraperitoneally near the abdominal midline to release a constant rate (0.25 μl/h) for 28 days. Twenty-eight days later, mice were anesthetized and a new minipump was surgically replaced for additional 4 weeks. Western-type diets were given to mice from the day of minipump implantation for 8 weeks.

### 2.3 *En face* detection of atherosclerotic lesions

Entire arterial trees were harvested from the level above coronary artery at the base of heart near the atria until 2–3 mm below the iliac bifurcation of the abdominal aorta. The arterial trees were fixed with 5% sucrose in 4% paraformaldehyde-PBS solution overnight. Perivascular fat pads were carefully removed. The arterial trees were pinned on the black wax-filled Petri dish and cut along the outer curvature of aorta from aortic root through innominate, carotid, and subclavian arteries until the iliac bifurcation of the abdominal aorta. After wash with PBS, arterial trees were stained with Sudan IV for 6 min, washed with 70% ethanol at room temperature for 3 min twice and images were taken using an iPhone. Area of arterial lesion detected by Sudan IV staining and area of entire arterial tree were measured by Image-Pro Plus 6.0 manually. Lesion area divided by area of total arterial tree and timed 100 was represented as the percentage of arterial lesions.

### 2.4 Biochemical analysis

Sera were collected from overnight fasting animals. Serum triglycerides (TG) and total cholesterol were measured by DRI-CHEM system (NX500i, FUJUFILM, Tokyo, Japan). ELISA kits were used to detect HDL-C (#79990), LDL-C (#79980; both from Crystal Chem, IL, USA), malondialdehyde (MDA, STA-832), and 4-HNE (STA-838; both from Cell Biolabs, CA, USA).

### 2.5 Quantitative reverse transcription-PCR

Total RNA was extracted from human aortic enfdothelial cells (HAECs) using the RNeasy Plus mini kit (#74134, Qiagen, Hilden, Germany). The first strand cDNA was synthesized from 1 μg of total RNA using the SuperScript III First-Strand Synthesis System kit (#18080-051, Invitrogen, MA, USA). Real-time PCR were amplified with primers specific for 3-hydroxy-3-methylglutaryl-CoA reductase (HMGCR), sterol regulatory element-binding protein-1 (SREBP1), SREBP2, proprotein convertase subtilisin/kexin type 9 (PCSK9), LDLR, HO-1, NQO-1, and β-actin using iQ SYBER Green Supermix reagent (#1708880, Bio-Rad, CA, USA) and detected by Step One Plus real-time PCR system (Applied Biosystems, MA, USA). Primers are listed at Table 1. Data were analyzed with iQ5 optical system software, Version 2.0 (Bio-Rad). Relative mRNA levels were normalized with the corresponding levels of β-actin.

**Table 1:** Primer sequences used for qRT-PCR.

Gene	5′ to 3′	Primers
β-actin	Sense	CACCATTGGCAATGAGCGGTTC
	Antisense	AGGTCTTTGCGGATGTCCACGT
HMGCR	Sense	TGCACCATGCCATCGATAGA
	Antisense	GGATTGTCTTTGCATGCTCCTT
HO-1	Sense	CTGTGAACTCTGTCCAATG
	Antisense	AACTGTGTCAGGTATCTCC
LDLR	Sense	AACCATTTTGGAGGATGAGAACC
	Antisense	CGTGAGTCGATTGGCACTGA
NQO-1	Sense	ATGAAGGAGGCTGCTGTAG
	Antisense	AGATGACTCGGAAGGATACTG
PCSK9	Sense	CATGGGATCTCAGGTCCTTCA
	Antisense	CACGCTGTAGGCTCCCAGA
SREBP1	Sense	CGACTACATCCGCTTCTTGCAG
	Antisense	CCTCCATAGACACATCTGTGCC
SREBP2	Sense	GCGTTCTGGAGACCATGGA
	Antisense	GGAACTCTCCCACTTGATTGCT

### 2.6 Cell adhesion assay

HAECs (7.5 × 10^4^ cells) were grown in 6-well plates at 37 °C. Cells were pretreated with FANC (100 nM) for 72 h and followed with ox-LDL (100 μg/ml) treatment overnight. THP-1 cells were suspended at a density of 1.0 × 10^6^ cells/ml with 0.1% BSA/Hank's Balanced Salt Solution (HBSS) and labeled with 1 μM of calcein-AM (#30026, Biotium, CA, USA) for 60 min at 37 °C. To perform adhesion assay, both HAECs and THP-1 cells were washed three times with 0.1% BSA/HBSS and then co-cultured together for 2 h at 37 °C. Nonadherent cells were removed carefully by three-time washings with 0.1% BSA/HBSS. Images of THP-1 cells attached on HAECs were acquired by applying inverted fluorescence microscopy (Leica, Wetzlar, Germany) at × 50 magnification and analyzed by Leica QWin image analysis software (Version number: V3.5.2, Cambridge, UK) to determine the average number.

### 2.7 Cholesterol efflux assay

THP1 cells were maintained at (2 x 10^5^/ml) in RPMI 1640 medium supplemented with 10% FCS. THP1 cells (2 x 10^5^/ml) were seeded in 24-well plates and differentiated using 200 nM phorbol 12-myristate 13-acetate (PMA, P8139-1 MG, Sigma-Aldrich, MO, USA) for 3 days. THP1-derived macrophages were loaded with fluorescent-labeled cholesterol (λ_Ex_ = 485/_λEm_ = 523 nm) buffer at 37 °C for 1 h. Following labeling, cells were incubated with serum free equilibration mix containing FANC (100 nM) and ox-LDL (100 μg/ml) for 16 h, followed by incubation with HDL or ApoA1 for 4 h. The fluorescence intensity of the medium and cell lysates were detected by fluorometry (Thermo Fisher Scientific, Waltham, USA) using 469 nm excitation and 537 nm emission filters. The cholesterol efflux was expressed as the fluorescence intensity in the medium /(the fluorescence intensity in medium + the fluorescence intensity in the cell lysate) according to the instructions of the manufacturer (MAK192, Sigma-Aldrich, MO, USA).

### 2.8 Immunofluorescence

To observe NRF2 nuclear entry, HAECs were seeded on 1% gelatin-coated 12-mm diameter cover slides (#41001112, Deckgläser, TH. GEYER, Württemberg, Germany) for 2 days. Cells were fixed with EM grade 4 % paraformaldehyde (157-8, Electron Microscopy Sciences, PA, USA). Cells were blocked with 10 % of horse serum and incubated with anti-NRF2 antibodies (16396-1-AP, proteintech, IL, USA) at 4°C overnight. Cells were incubated with secondary antibodies at room temperature for 3 h and counter stained with DAPI. Slides were filled with ProLong™ diamond antifade mountant (P36970, Thermo Fisher Scientific, MA, USA). Images were acquired under Leica SP8 microscopy.

Intestinal tissues were washed in PBS and fixed in 4 % paraformaldehyde (158127-500G, Sigma-Aldrich, MO, USA) for 4 h at 4°C before preparation of 6-μm cryosections. Intestinal tissues were incubated with 50 % Tissue-Tek® O.C.T. Compound (#4583, Sakura Finetek, CA, USA) in 15% sucrose-PBS for 16 h at 4°C with gentle rotation and then followed with 100% OCT for 2 h at 25 °C. Tissues were snap frozen in liquid nitrogen and stored at -80°C. Tissue slides were fixed with 4% paraformaldehyde at room temperature for 10 min. After 1 h of blocking with 10% of horse serum to reduce nonspecific binding, tissues were incubated with primary antibodies at 4 °C overnight. Antibodies against Niemann-Pick C1-Like 1 (NPC1L1) (#PA1-16800, invitrogen, MA, USA) and Villin (sc-58897, Santa Cruz, CA, USA) used at a dilution of 1: 100. Tissues were incubated with secondary antibodies at room temperature for 3 h and counter stained with DAPI. Slides were filled with ProLong™ diamond antifade mountant (P36970, Thermo Fisher Scientific, MA, USA). Images were acquired under Leica SP8 confocal microscopy (DMi8, Leica, Wetzlar, Germany).

For filipin staining, the protocol was essentially the same as described [29] that 4% PFA-fixed tissue sections were incubated with 1.5 mg/ml glycine in PBS for 30 min, then incubated with filipin (125 μg/ml) in PBS for 1 h at room temperature. After three washes, the tissue sections were mounted with ProLong Anti-Fade media before image processing.

### 2.9 Immunoprecipitation and western blotting

Cells were lysed with RIPA Buffer (ab156034, abcam, Cambridge, UK) with Halt Protease Inhibitor Cocktail (78438, Thermo Fisher Scientific, MA, USA). Protein concentrations were determined by modified Lowry's method and adjusted to 1 mg/ml for immunoprecipitation. Fifty μg (5%) of lysates were kept for ‘input’ for immunebolted with indicated antibodies. Primary antibodies were incubated with protein A/G agarose at room temperature for 2 h. Antibodies (IgG) were cross-linked with Protein A/G agrose beads by N-Ethyl-N′-(3-dimethylaminopropyl) carbodiimide (EDC) (#03450-1G, Sigma-Aldrich, MO, USA). Immunoprecipitation was performed under 1 ml of cell lysates with 1 μg of antibody-conjugated agarose beads rotated at 4°C overnight. After 3 times of RIPA washes, the immunocomplexes were eluted by indicated reagents or 1x Laemmli solution. Whole-cell lysates and samples from immunoprecipitation were subjected to SDS-polyacrylamide gel electrophoresis (SDS-PAGE) and the separated proteins were transferred to nitrocellulose blots electrophoretically. The blots were blocked with 10% bovine serum albumin and probed with indicated antibodies. Information of antibodies is described in Table 2. Immunoblots were detected by enhanced chemiluminescence method (GERPN2236, Sigma-Aldrich, MO, USA), according to the instructions of the manufacturer.

**Table 2:** Antibodies used for Western blot and immunoprecipitation.

Antibody	Clone	Host	Manufacturer	Catalog#	Dilution
ABCA1	AB.H10	Mouse	abcam	ab18180	1:1000
AKT	11E7	Rabbit	Cell Signaling	#4685	1:1000
*p*-AKT	D9E	Rabbit	Cell Signaling	#4060	1:1000
HO-1	Polyclone	Rabbit	proteintech	10701-1-AP	1:1000
ICAM-1	Polyclone	Rabbit	Cell Signaling	#4915	1:1000
KEAP1	D6B12	Rabbit	Cell Signaling	#4915	1:1000
LDLR	Polyclone	Rabbit	proteintech	10785-1-AP	1:1000
Lipoic acid	Polyclone	Rabbit	Merck	437695	1:1000
NPC1L1	Polyclone	Rabbit	Invitrogen	# PA1-16800	1:1000
NRF2	Polyclone	Rabbit	proteintech	16396-1-AP	1:1000
Ubiquitin	Polyclone	Rabbit	abcam	ab7780	1:1000
VCAM-1	E1E8X	Rabbit	Cell Signaling	#13662	1:1000
Villin	1S2C3	Mouse	Santa Cruz	sc-58897	1:1000

### 2.10 Statistics

Continuous data were expressed as mean ± SD and compared with two-tailed *t*-test. For multiple comparisons, data were analyzed by one-way ANOVA followed by Newman-Keuls comparison test. All p values were two-sided with a value less than 0.05 considered statistically significant.

## 3 Results

### 3.1 Antioxidant and anti-inflammatory potency of FANC compared to AuNPs and α-lipoic acid

To clarify the contribution of FANC and the component AuNPs and α-lipoic acid to the antioxidant and anti-inflammatory properties, comparisons were made among the three molecules based on the amount of AuNPs and α-lipoic acid contained in FANC. We first tested the antioxidant effects by lipopolysaccharide (LPS)-induced ROS production in HAECs (Figs. S1A and S1B). For this purpose, 100 nM FANC, 8 μM DHLA, and 25 μM AuNP were used (please refer to subsection “Composition of gold and DHLA in FANC” in Supplementary Methods for the calculation of components of FANC). The results showed that 100 nM FANC and 25 μM AuNP exhibited antioxidant effects significantly, while 8 μM DHLA was not significantly effective. We then, using the same concentrations of the three molecules, tested the effects on LPS-induced pro-inflammatory molecules. As shown in Figs. S1C–S1F, only 100 nM FANC alleviated LPS-induced ICAM-1 and IL-8 significantly and both 100 nM FANC and 25 μM AuNP alleviated VCAM-1 and IL-6 significantly, while 8 μM DHLA was not effective. Therefore, FANC enhanced the antioxidant and anti-inflammatory activities of the component DHLA.

### 3.2 FANC alleviate chow diet and western-type diet-induced atherosclerotic lesions and decrease total cholesterol in hypercholesterolemic mice

**Fig. 1:**
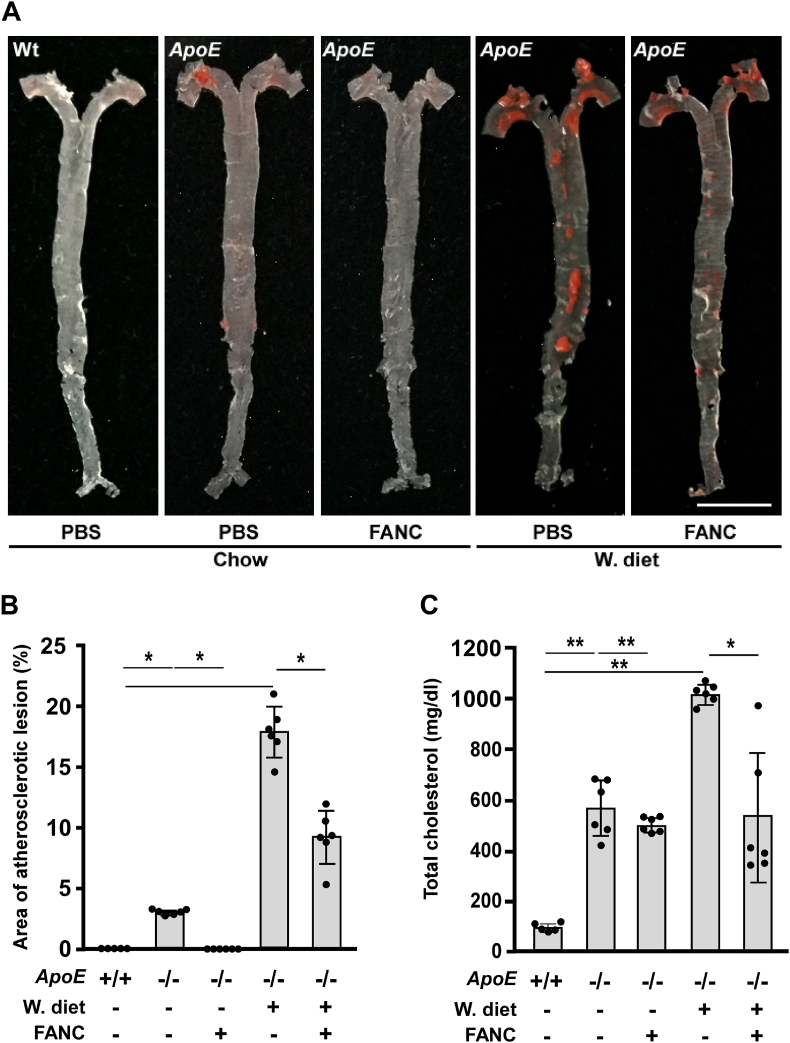
Effects of FANC on atherosclerotic lesions and serum total cholesterol in *ApoE*-deficient mice fed different diets (A) Arterial trees of wild type (Wt) and *ApoE-*deficient (*ApoE*^*-/-*^) mice treated with PBS or FANC (20 μM) intraperitoneally via osmotic minipumps at a constant rate (0.25 μl/h) and fed chow or Western-type diet (W. diet) for 8 weeks were stained with Sudan-IV (red) to show atherosclerotic lesions. n = 5 for Wt and n = 6 for each *ApoE*^*-/-*^ mice group (*i.e.* n = 6, 6, 6, 6 for chow, FANC, W. diet, W. diet + FANC, respectively). Scale bar, 0.5 cm. (B) Quantification of atherosclerotic lesion area in arterial trees. (C) Effects of FANC on *W*. diet-induced hypercholesterolemia. Sera were collected at the end of experiment. ∗, p<0.05; ∗∗, p<0.01. (For interpretation of the references to colour in this figure legend, the reader is referred to the Web version of this article.)

Since atherosclerosis is classified as chronic inflammation, the anti-atherosclerotic effects of FANC were tested. *ApoE-*deficient mice fed normal chow developed atherosclerotic lesions and the lesions were much more in those fed Western-type diet, while wild type, C57BL/6J mice were free of the lesions (Fig. 1A and B). FANC were given by osmotic minipumps to release 20 μM concentration at a constant rate of 0.25 μl/h. FANC effectively ameliorated atherosclerotic lesions both in chow and Western-type diet-fed *ApoE*-deficient mice (Fig. 1A and B). Blood test showed serum total cholesterol levels were correlated with the dimension of atherosclerotic lesions and the levels were reduced by FANC treatment (Fig. 1C).

### 3.3 Effects of different FANC dosage and administration routes on western-type diet-induced atherosclerotic lesions, lipid profile changes, lipid peroxidation and hepatic lipid accumulation in hypercholesterolemic mice

**Fig. 2:**
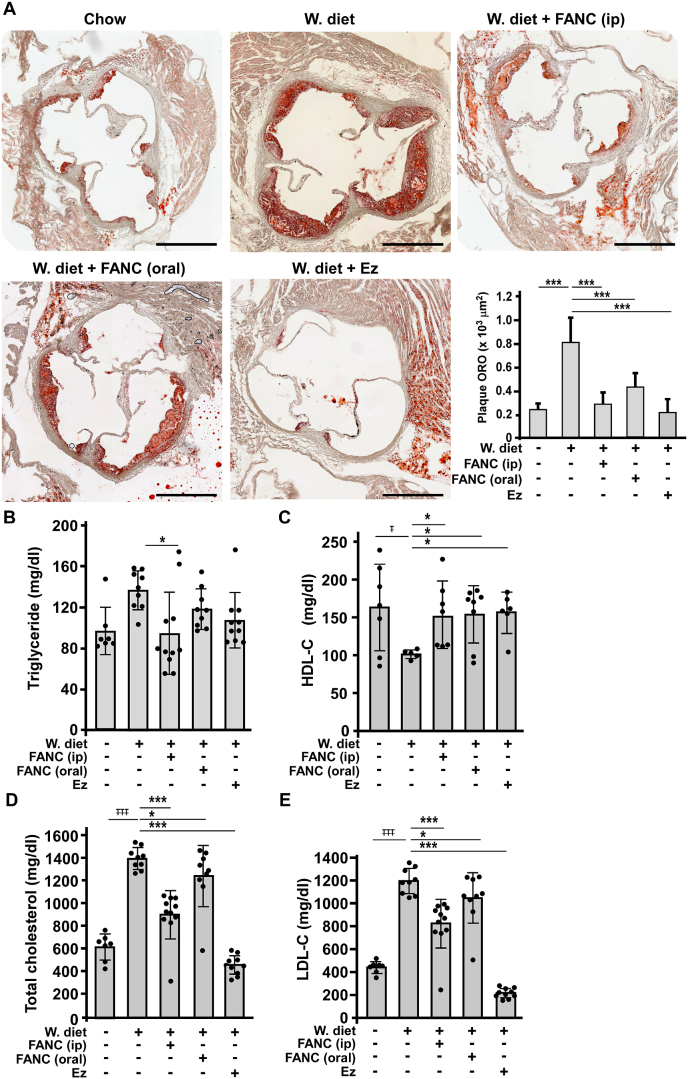
Comparison of FANC administration routes on Western-type diet-induced atherosclerotic lesions and lipid profile changes in *ApoE*-deficient mice *ApoE*-deficient (*ApoE*^*-/-*^) mice concomitantly fed Western-type diet (W. diet) and treated with FANC intraperitoneally (FANC (ip)) or orally (FANC (oral) for 8 weeks were compared. Mice treated with Ezetimibe (Ez, 0.005%, w/w) were used as control. (A) Aortic sinuses were sectioned and stained with oil-red O. The signal appearing in plaque areas were measured and quantified. Representative images were sections from 3 aorta roots of each group (n = 3, 3, 3, 3, 3 of chow, W. diet, W. diet + FANC (ip), W. diet + FANC (oral), W. diet + Ez), Scale bar, 500 μm. (B) to (E) Effects of different FANC administration routes on W. diet-induced lipid profile changes. Sera were collected at the end of experiments. n = 7 for chow-fed mice, n = 9 for W. diet-fed animals, n =11 for FANC (ip), n = 9 for FANC (oral) and n = 10 for Ez group. ∓, compared with chow diet; ∗, compared with W. diet; ∓ and ∗, p<0.05; ∗∗, p<0.01; ∓∓∓ and ∗∗∗, p<0.001. (For interpretation of the references to colour in this figure legend, the reader is referred to the Web version of this article).

We also tested the effects of different dosage of FANC by oral administration on atherosclerotic lesions and serum cholesterol. As Fig. S2 shows, a train of FANC at 30, 100 and 300 nM in drinking water showed that FANC decreased atherosclerotic lesions and serum cholesterol in a dose-dependent manner, reaching statistical significance at 300 nM (p<0.05). Thereafter, 300 nM of FANC in the drinking water (FANC (oral)) was compared with FANC via osmotic minipumps (FANC (ip)) (Fig. 2). Consistently, FANC given by both routes effectively decreased atherosclerotic lesions in aortic sinuses (Fig. 2A) and arterial trees (Fig. S3A). In addition to anti-atherosclerotic effects, FANC ameliorated Western-type diet-induced pro-inflammatory responses profoundly (Figs. S3B and S3C). Ezetimibe, a cholesterol-lowering drug on the market, was given using customer specified Western-type diet (0.005% w/w) to compare its effects with FANC. Sera were harvested to examine lipid profile changes, including total cholesterol, triglycerides, high-density lipoprotein cholesterol (HDL-C), and LDL-C. Consistent with previous findings, triglycerides levels were not significantly affected by Western-type diet in *ApoE*-deficient mice (Fig. 2B, [30]), while intraperitoneal rout of FANC reduced the level. Serum level of HDL-C was significantly reduced by Western-type diet but the reduction nearly disappeared in both FANC and Ezetimibe treatment groups (Fig. 2C). Both routes of FANC treatment decreased total cholesterol and LDL-C in *ApoE-*deficient mice fed Western-type diet (Fig. 2D and E), though there was a trend that atherosclerotic lesions were reduced more by FANC given intraperitonealy, compared to FANC given orally. In the parallel study, the effects of FANC on Western-type diet-induced atherosclerotic lesions and cholesterol lowering were further confirmed in *LDLR*-deficient mice (Fig. S4). Both routes of FANC treatments recaptured the therapeutic effects on atherosclerotic lesions and the decrease of total cholesterol and LDL-C shown in *ApoE-*deficient mice. As Western-type diet induced lipid profile changes in bloods, we further examined its impacts in liver. Lipid deposits in liver were examined by Oil-red O staining. As shown in Fig. S5A, Western-type diets profoundly increased lipid accumulation in liver that was almost rescued by Ezetimibe and FANC (ip) treatments, but only partly alleviated by FANC (oral) treatment. The results of total cholesterol extracted from liver tissue also showed similar changes (Fig. S5B). Hypercholesterolemia was reported to increase ROS, leading to enhanced production of MDA and 4-HNE. The antioxidant property of FANC [12,13] *in vivo* was further confirmed in reduction of serum reactive aldehydes MDA and 4-HNE (Figs. S5C and S5D). The organ and tissue levels of FANC given by different delivery routes varied widely in the blood and liver, but not heart (Fig. S5E).

### 3.4 Mechanisms underlying lipid-lowering effects of FANC

**Fig. 3:**
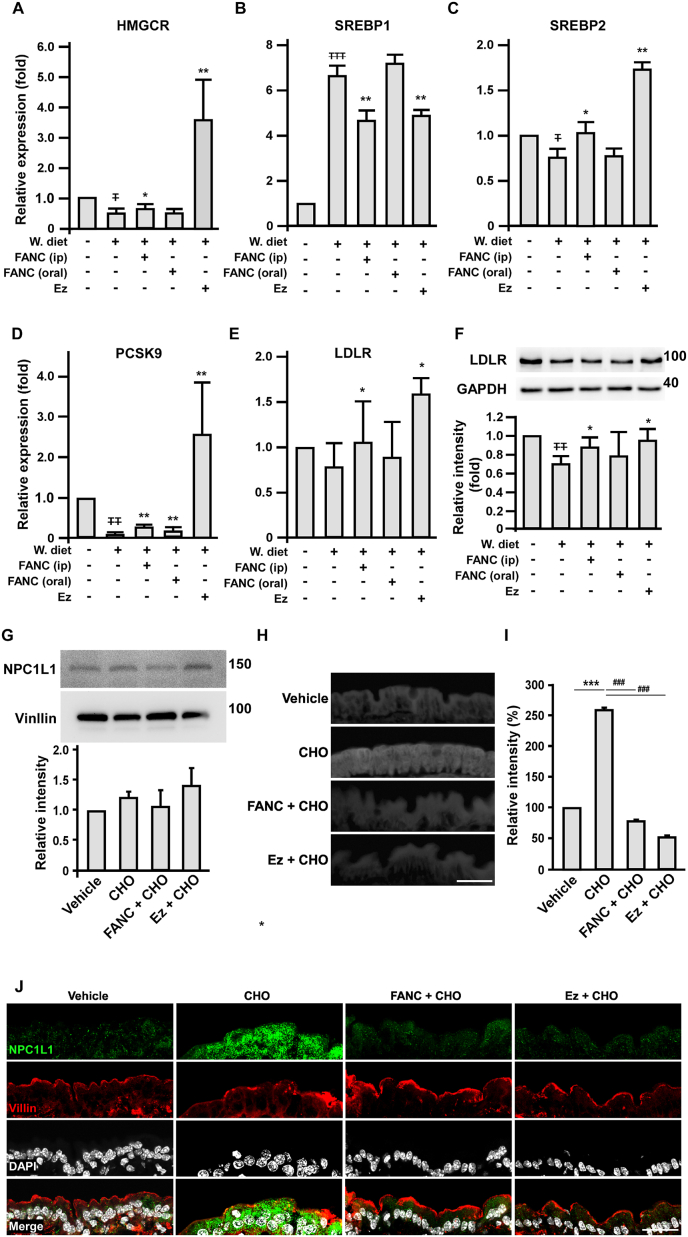
Effects of FANC on cholesterol homeostasis-related genes and proteins in hypercholesterolemic mice and on intestinal cholesterol absorption in wild-type mice (A) to (E), Quantitative PCR (qPCR) of relative expression of HMGCR, SREBP1, SREBP2, PCSK9 and LDLR in livers of Western-type diet fed ApoE deficient mice with indicated treatments. n = 7 for chow-fed mice, n = 9 for W. diet-fed animals, n =11 for FANC (ip), n = 9 for FANC (oral) and n = 10 for Ez group. (F) Western blot analysis of LDLR protein from liver extracts. Values are mean ± SD of triplicate assays from 3 independent experiments. ∓, compared with chow diet; ∗, compared with W. diet; ∓ and ∗, p<0.05; ∓∓ and ∗∗, p<0.01; ∓∓∓, p<0.001. (G) NPC1L1 protein expression and (H) sections of intestine from FANC or Ezetimibe pretreated wild-type mice at 1 h post cholesterol feeding and stained with filipin to detect cholesterol absorption. Six mice were assigned for each group. Scale bar, 50 μm. (I) Quantification of intestinal filipin staining. n=3. (J) Effects of FANC and Ezetimibe on cholesterol-mediated NPC1L1 distribution subjacent to brush border. Tissue sections were treated with antibodies against NPC1L1 (green) and Villin (red) to mark brush border, and with DAPI (gray) for nuclei staining. n=3 for each group. CHO, cholesterol. ∗, compared with vehicle; #, compared with CHO group; ∗∗∗ and ###, p<0.001. (For interpretation of the references to colour in this figure legend, the reader is referred to the Web version of this article.)

The expressions of cholesterol homeostasis-related genes in liver were determined by quantitative PCR (qPCR) and western blotting to assay the expression of HMGCR, SREBP 1, SREBP 2, PCSK9, and LDLR (Fig. 3A to E). FANC (ip) attenuated the changes in expression of these sterol-regulated genes in response to Western-type diet (p<0.05 for HMGCR, SREBP1 and LDLR; p<0.01 for SREBP2 and PCSK9, similar to Ezetimibe, which attenuated or even reversed the changes, while FANC (oral) only rescued PCSK9 expression (p < 0.01). The similarity also applied to LDL receptor protein expression. Of note, Ezetimibe in the Western-type diet group remarkably altered these sterol-regulated genes that had been reported [31].

The trend of changes of hepatic cholesterol homeostasis-related genes and protein in the mice treated with FANC resembling those treated with Ezetimibe prompted us to examine the effects of FANC on intestinal cholesterol absorption. NPC1L1, a transporter mainly located at the apical membrane (brush border) of intestine, is responsible for the clathrin-mediated endocytosis of cholesterol [32,33]. Ezetimibe prevents cholesterol absorption by impairing the endocytosis of NPC1L1, leading to the accumulation of NPC1L1 at the apical membrane [34,35]. The overall NPC1L1 proteins harvested from lumen of duodenum were not affected with FANC or Ezetimibe pretreatment, or cholesterol feeding, indicating that during the experiment period, the expression of NPC1L1 remained stationary (Fig. 3G). Indeed, Ezetimibe as well as FANC markedly prevented intestinal cholesterol absorption in wild-type mice (Fig. 3H and I). NPC1L1 was readily available at the apical membrane of small intestine of animals after 1 h of cholesterol feeding, indicating its role in cholesterol absorption (Fig. 3J). In animals with Ezetimibe or FANC pretreatments, the levels of apical NPC1L1 were close to that of vehicle group, suggesting their inhibiting effects on intestinal NPC1L1-mediated cholesterol absorption (Fig. 3J).

### 3.5 FANC activate NRF2-mediated HO-1 and NQO-1 expression

**Fig. 4:**
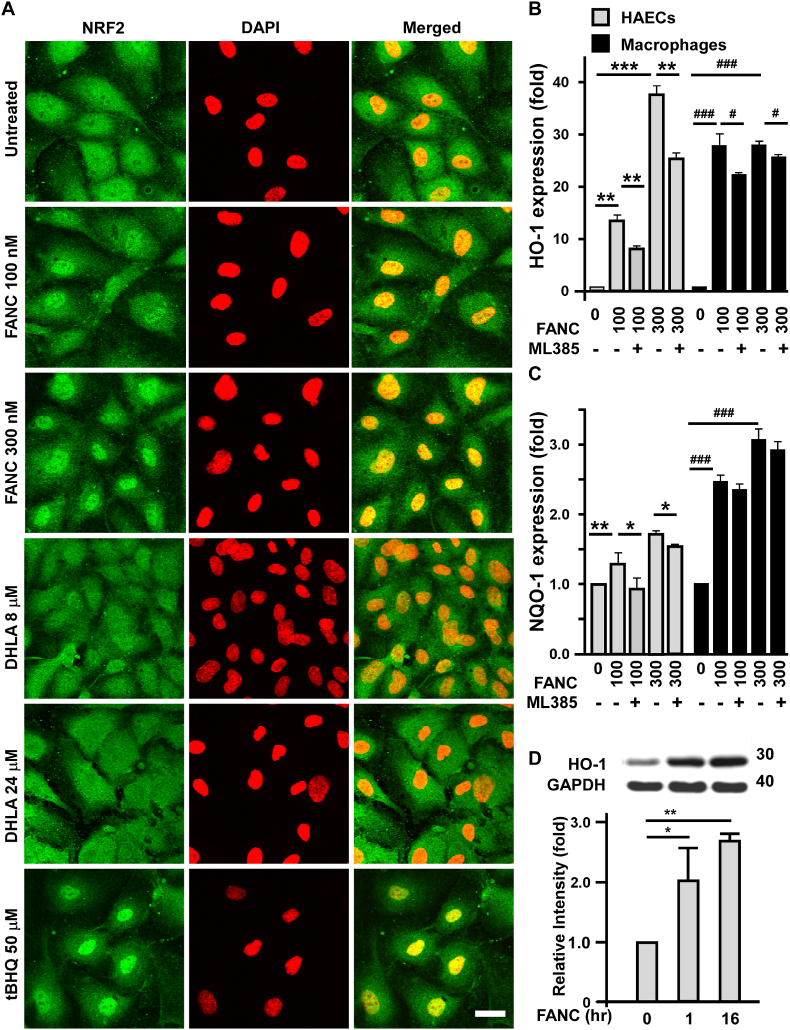
FANC activate NRF2-mediated HO-1 and NQO-1 expression (A) Activation of NRF2 (green labels) by FANC. HAECs were treated with indicated concentration of FANC (nM), DHLA (μM) or tBHQ (μM) for 6 h. Cells were stained with anti-NRF2 antibodies and DAPI for nuclear staining (red labels). Scale bar, 50 μm. (B) and (C) ML-385 inhibited FANC-induced HO-1 and NQO-1 transcription. HAECs and macrophages were treated with ML-385 (10 μM) for 1 h and then FANC for 4 h followed by qPCR analysis. (D) FANC induced HO-1 expression. HAECs were treated with FANC (100 nM) for 1 and 16 h followed by Western blot assay. Values are mean ± SD of triplicate assays from 3 independent experiments. ∗ and #, p<0.05; ∗∗, p<0.01; ∗∗∗ and ###, p<0.001. (For interpretation of the references to colour in this figure legend, the reader is referred to the Web version of this article.)

Regarding the effects of FANC on decreasing LPS-induced ROS (Fig. S1B) and Western-type diet-induced reactive aldehydes (Figs. S5C and S5D), we wondered whether FANC activated NRF2, a key regulator of the electrophile counter-attack excess ROS for the production of dysregulated lipid peroxides. To this end, HAECs were treated with tert-butylhydroquinone (tBHQ), an inducer of KEAP1-NRF2 system. tBHQ increased the level of NRF2 and promoted its nuclear entry (Fig. 4A). A similar response was seen for NRF2 to FANC, and showed a dose-sensitive manner. The same molality of capped DHLA were tested, however, DHLA did not promote NRF2 nuclear translocation up to 24 μM. The expressions of NRF2 target genes, HO-1 and NQO1 were induced by FANC in both HAECs and THP-1-induced macrophages, while ML385, a NRF2 inhibitor, inhibited the induction significantly in HAECs, but mildly in THP-1-induced macrophages (Fig. 4B and C, by qPCR). The induction was not seen in vascular smooth cells (data not shown). The duration of HO-1 protein induction by FANC persisted up to 16 h in HAECs (Fig. 4D).

### 3.6 FANC interfere the binding of KEAP1 with NRF2 and promote KEAP1 degradation

**Fig. 5:**
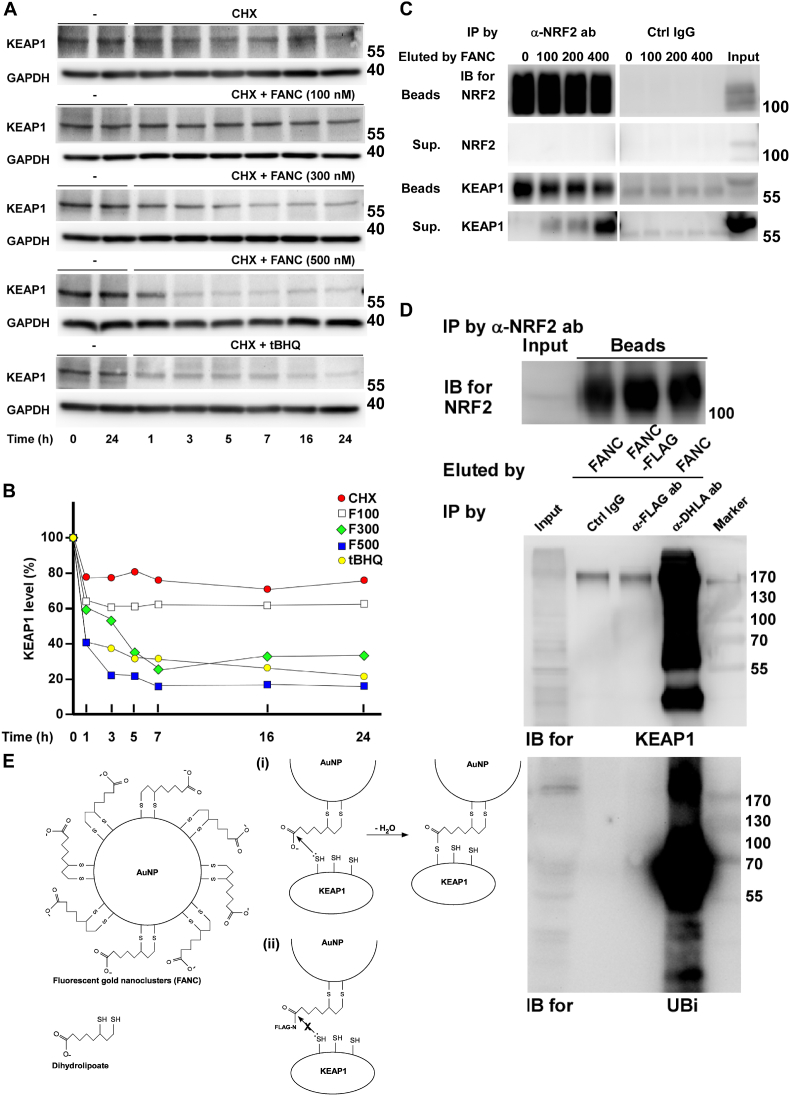
FANC promote KEAP1 degradation and release from NRF2 immunocomplexes (A) Effects of FANC on KEAP1 stability. HAECs were treated with indicated concentrations of FANC or tBHQ (50 μM) in the presence of cycloheximide (CHX, 100 μg/ml). Cells were harvested at the indicated time points and immunoblots were probed with anti-KEAP1 antibodies. (B) Quantification of relative level of KEAP1. Band intensities of KEAP1 at various time points were quantified and normalized to those without CHX treatment (set as 100% at t_0_). (C) FANC promoted KEAP1 eluted from NRF2 immunocomplexes in a dose-dependent manner. NRF2 immunocomplexes were incubated with indicated concentrations (nM) of FANC. Proteins either in immunocomplexes (beads) or in supernatants (Sup.) were identified by anti-NRF2 and anti-KEAP1 antibodies, respectively. (D) Determination the interaction between FANC and KEAP1 by tandem immunoprecipitation. At the first run of immunoprecipitation, NRF2 was pulled down by anti-NRF2 antibodies and eluted by FANC or Flag-tagged FANC (FANC-FLAG). Equal amount of NRF2 were left in beads (upper panel). At the second run of immunoprecipitation, FANC or FANC-FLAG eluates were either pulled down by control IgG (Ctrl IgG), anti-FLAG antibodies (α-FLAG ab) or anti-DHLA antibodies (α-DHLA ab). The immunoblot was probed with anti-KEAP1 antibodies (middle panel). Lower panel, the duplicated immunoblot as middle panel was probed with anti-ubiquitin (UBi) antibodies. Consistent results were obtained from three independent experiments. (E) Scheme explains thioesterification occurred between KEAP1 and FANC (i), while the reaction was blocked by FLAG-tagged FANC (ii).

At resting state, constitutive expression of NRF2 is ‘clamped’ by KEAP to degrade at proteasome in Cul3-mediated ubiquitination. In the presence of antioxidants, the cysteine residues of KEAP1 are reduced to make NRF2 available for nuclear entry [19]. Therefore, we tested a series concentration of FANC on the stability of KEAP1. Cells were pretreated with cycloheximide to inhibit protein synthesis to study the half-life of interested targets. As Fig. 5A and B showed, the degradation curves of KEAP1 moved downwardly with the increased concentrations of FANC, suggesting that FANC promoted KEAP1 degradation. Next, we wondered if FANC interfere the binding of KEAP1 with NRF2. To verify this assumption, we conducted a competition binding assay of FANC in the immunocomplexes of NRF2-KEAP1 (Fig. 5C). The level of eluted KEAP1 from the immunocomplexes by FANC in supernatant (Sup) increased in a dose-dependent manner, while KEAP1 left in the immunocomplexes (beads) was inversely decreased, arguing that FANC promoted KEAP1 eluted from NRF2-KEAP1 immunocomplexes. The level of NRF2 left in beads was not affected by FANC dosages and not presented in Sup, suggesting that FANC interfere the interaction of NRF2 and KEAP1. To further explore this finding, we performed two runs of immunoprecipitation simultaneously (Fig. 5D). At first, NRF2 was pulled down by anti-NRF2 antibodies (Fig. 5D, upper panel) and eluted by FANC or FLAG-tag FANC. At the second run of immunoprecipitation, the eluted FANC solutions were incubated with control IgG or anti-DHLA IgG beads, and the eluted FLAG-tag solutions were incubated with anti-FLAG IgG beads. By Western blot assay, KEAP1 was detected in anti-DHLA IgG pulldown of FANC eluents, indicating the binding of KEAP1 with FANC, while KEAP1 was not detected in anti-FLAG IgG pulldown of FLAG-tagged FANC eluents (Fig. 5D, middle panel). Of note, the comet tail of KEAP1 suggested its ubiquitination, which was confirmed by anti-ubiquitin antibodies (Fig. 5D, lower panel).

As FLAG-tagged FANC prevented KEAP1 eluted from the NRF2 immunocomplexes, the result might suggest that the capped ligand DHLA, when free of FLAG tag, bound KEAP1 and the pulldown by anti-DHLA beads contained KEAP1 (pathway (i), Fig. 5E for scheme explanation), while DHLA in FANC with FLAG tag lost the ability to bind KEAP1 and the pulldown by anti-FLAG IgG beads was free of KEAP1 (pathway (ii), Fig. 5E).

### 3.7 FANC activate IRβ-AKT signaling and HO-1-mediated suppression of pro-inflammatory adhesion molecule expression in HAECs

**Fig. 6:**
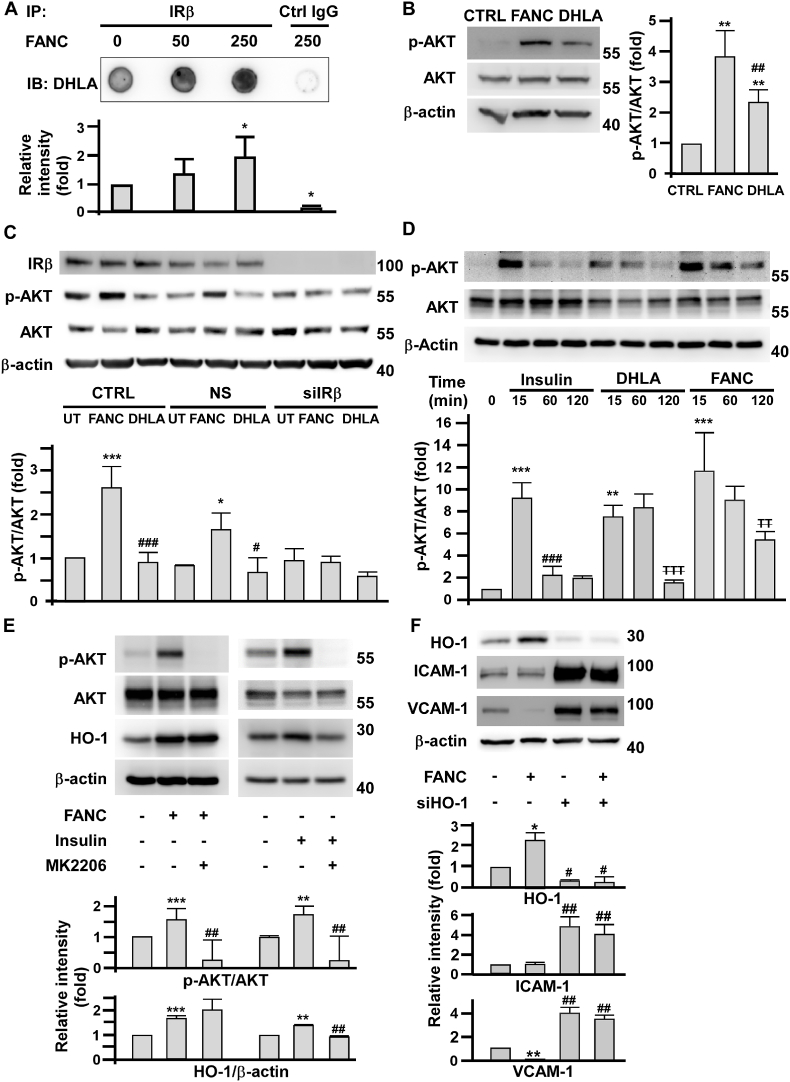
Effects of FANC and insulin on AKT activation and pro-inflammatory adhesion molecule expression in human aortic endothelial cells (A) Dot blot assay to determine the binding of FANC to IRβ. Immunoprecipitated anti-IRβ and control IgG were incubated with indicated concentrations (nM) of FANC. The pull-down FANC were detected by anti-DHLA antibodies. Lower panel, quantification of relative chemiluminescence from three independent experiments. ∗, p<0.05 compared to the leftmost bar. (B) Effects of FANC and DHLA on AKT activation. HAECs were respectively treated with 100 nM of FANC and 8 μM of DHLA for 30 min. Activation of AKT was determined and quantified by the ratio of *p*-AKT/AKT. ∗∗, p<0.01 compared with control. ##, p<0.01 compared with FANC group. (C) Effects of IRβ depletion on FANC and DHLA-mediated AKT activation. HAECs were transfected with nonsense (NS) or IRβ silencing RNA (siIRβ) for 16 h. Cells were treated with FANC or DHLA for 2 h and harvested to assay the expression of IR-β and AKT activation. UT, untreated cells. ∗, compared with UT; ^#^, compared with FANC group. ∗ and ^#^, p<0.05; ∗∗∗ and ^###^, p<0.001. (D) Activation kinetics of AKT under insulin, DHLA, and FANC treatment. HAECs were treated with insulin (100 nM), DHLA (8 μM) or FANC (100 nM) to determine the ratio of *p*-AKT/AKT. ∗, compared with t_0_, ^#^, compared with t_15_, ^T^, compared with t_60_, ∗∗ and ^TT^, p<0.01. ∗∗∗, ^###^ and ^TTT^, p<0.001. (E) Effects of MK2206 on FANC and insulin-mediated AKT activation and HO-1 induction. HAECs were pretreated with MK2206 (1 μM) for 16 h. Lysates were harvested after 15 min of FANC or insulin treatments to determine AKT activation. For HO-1 induction, cells were collected after 6 h of FANC or insulin treatment. ∗, compared with UT; #, compared with FANC group. ∗∗ and ##, p<0.01.∗∗∗, p<0.001. (F) Effects of HO-1 depletion on FANC-mediated ICAM-1 and VCAM-1 expression. HAECs were transfected with HO-1 silencing RNA (siHO-1) for 16 h, followed by treatment with 100 nM FANC for 16 h. Cells were harvested to assay the expression of HO-1, ICAM-1 and VCAM-1. Values are mean ± SD of triplicate assays from 3 independent experiments. ∗, compared with UT; #, compared with FANC only group. ∗and #, p<0.05; ∗∗ and ##, p<0.01.

Alpha-lipoic acid had been reported in computer modeling studies to possess a direct binding site at the tyrosine kinase domain of the IRβ [36]. As DHLA is the capped ligand of FANC, we tested the effects of FANC on activating IRβ-protein kinase B (AKT) signaling pathway. Firstly, the immunoprecipitated IRβ was incubated with FANC to perform dot blotting assay. The presence of FANC in the immunocomplexes was determined by anti-DHLA antibodies (Fig. 6A). The results did confirm existence of FANC in the immunocomplexes, suggesting that FANC bound IRβ via the capped DHLA. Secondly, it was known that the binding of DHLA to IRβ phosphorylates insulin receptor substrate [37] and activates AKT [38]. As shown in Fig. 6B, both FANC and the same molality of capped DHLA activated AKT. However, FANC were more powerful than DHLA to induce AKT phosphorylation (p<0.01). The requirement of IRβ for FANC and DHLA-mediated AKT activation was examined by IRβ siRNA depletion. As shown (Fig. 6C), the effects of FANC and DHLA on AKT phosphorylation were blunted by IRβ depletion. The activation kinetics of AKT by insulin, DHLA, and FANC treatments were examined in HAECs. FANC showed the strongest potency and last for more than 120 min, while the effects of insulin and DHLA nearly vanished before 120 min (Fig. 6D). Insulin manifested anti-inflammatory effect by inducing the expression of HO-1 to suppress the expression of VCAM-1 in endothelial cells [39,40]. Therefore, the role of insulin/AKT/HO-1 signaling axis in HAECs was examined. MK2206, an inhibitor of AKT, completely inhibited FANC and insulin-induced AKT activation (Fig. 6E). However, for HO-1 induction, FANC were thoroughly resistant to MK2206.

The requirement of HO-1 for FANC-mediated ICAM-1 and VCAM-1 suppression was examined by siRNA depletion. At the same concentration of 100 nM, FANC induced HO-1 expression with the suppression of VCAM-1 but not ICAM-1 (Fig. 6F). Interestingly, under HO-1 depletion, the suppression of ICAM-1 and VCAM-1 were completely lost in the presence of FANC, indicating the requirement of HO-1 in FANC-mediated anti-inflammatory responses.

### 3.8 FANC induce macrophage ABCA1 expression and ameliorate macrophage attachment onto endothelial cells

**Fig. 7:**
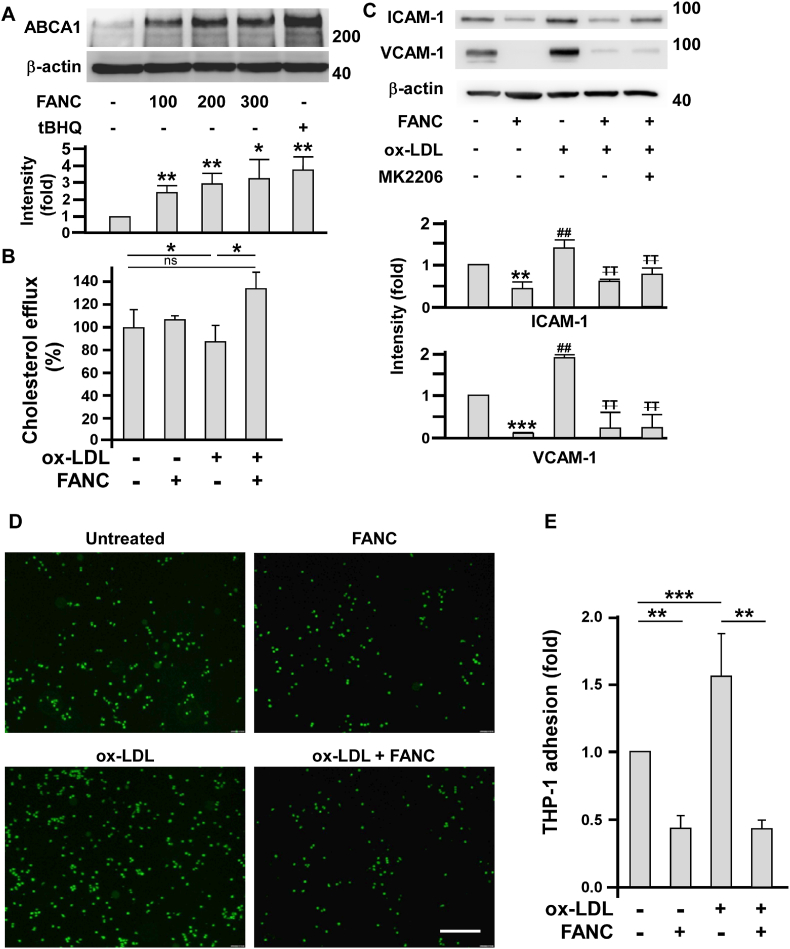
Effects of FANC on macrophage ABCA1 expression, cholesterol efflux, and ox-LDL-induced adhesion onto endothelial cells plus endothelial pro-inflammatiory adhesion molecule expression (A) FANC enhanced ABCA1 expression in THP-1-derived macrophages. Cells were incubated with indicated concentrations (nM) of FANC or tBHQ (100 nM) for 16 h. Compared with leftmost bar, ∗, p<0.05; ∗∗, p<0.01. (B) FANC enhanced cholesterol efflux. THP-1-derived macrophages were treated with FANC (100 nM) and ox-LDL (100 μg/ml) for 16 h. The fluorescence intensity of the medium and cell lysates was detected by fluorometry. ∗, p<0.05 (C) FANC attenuated ox-LDL-induced pro-inflammatory adhesion molecule expression. HAECs were pretreated with 100 nM of FANC or MK2206 (1 μM) for 24 h followed by treatment with 100 μg/ml of ox-LDL for 16 h ∗ and #, compared with UT; ∓, compared with ox-LDL only group. ∗∗, ^##^ and ∓∓, p<0.01; ∗∗∗, p<0.001. (D) FANC inhibited ox-LDL-induced macrophage adhesion onto HAECs. HAECs were pretreated with 100 nM FANC for 24 h, followed by treatment with 100 μg/ml of ox-LDL for 16 h. THP-1 derived macrophages were loaded with Calcein-AM (1 μM, green) and incubated with HAECs to perform adhesion assay. (E) Analysis of attached macrophage number on HAECs. ∗∗, p<0.01; ∗∗∗, p<0.001. Values are mean ± SD of triplicate assays from 3 independent experiments. (For interpretation of the references to colour in this figure legend, the reader is referred to the Web version of this article.)

The finding that FANC decreased Western-type diet-induced atherosclerotic lesions (Fig. 1, Fig. 2A) prompted us to ask whether FANC increased macrophage ABCA1 transporters to promote cholesterol efflux. Indeed, FANC enhanced macrophage ABCA1 expression (Fig. 7A). In functional assay, FANC resumed ox-LDL-impaired cholesterol efflux in macrophages (Fig. 7B). Hypercholesterolemia is associated with increased ox-LDL level, resulting in endothelial pro-inflammatory responses [41]. As expected, ox-LDL induced the expression of ICAM-1 and VCAM-1 in HAECs that were markedly alleviated by FANC treatment (Fig. 7C). In macrophage attachment assay, FANC ameliorated ox-LDL-induced macrophage attachment onto HAECs (Fig. 7D and E).

## 4 Discussion

This study showed that, in animal models of hypercholesterolemia and atherosclerosis, FANC reduced serum total cholesterol and LDL-C, and attenuated aortic atheroma burden. The animal models included *ApoE*-deficient mice and *LDLr*-deficient mice fed high fat diet. In *ApoE*-deficient mice, the oxidative stress markers MDA and 4-HNE were also lowered. The effects of FANC were achieved by oral or intraperitoneal delivery, respectively. The mechanisms underlying lipid-lowering effects involved inhibition of intestinal absorption of cholesterol. At molecular level, FANC was a ligand for IRβ and activated AKT in endothelial cells. However, unlike binding of insulin to IRβ to activate AKT and induce HO-1 expression, induction of HO-1 by FANC was independent of activation of AKT. Apart from the IRβ-AKT axis, FANC also activated NRF2 to induce HO-1 by uncoupling and degradation of KEAP1 from NRF2. At cellular level, FANC attenuated ox-LDL-induced expression of pro-inflammatory molecules ICAM-1 and VCAM-1, and inhibited macrophage adhesion onto endothelial cells. In addition, FANC enhanced ABCA1 expression in macrophages and cholesterol efflux in the cells stimulated by ox-LDL. All the properties worked coherently to contribute to the anti-atherosclerotic action of FANC.

### 4.1 FANC possess cholesterol-lowering effect

One novel finding of the present study is that FANC possessed lipid-lowering effects in the hyperlipidemic mice. AuNPs had been reported to potentiate the antihyperlipidemic effects of *Paeonia emodi* in poloxamer-407-induced hyperlipidemic mice [42]. However, to our knowledge, the components of FANC, DHLA did not lower cholesterol [43]. The present study showed that the lipid-lowering effects of FANC was attributable to the inhibition of intestinal cholesterol absorption, similar to the action of Ezetimibe, which, compared to FANC, exerted a better lipid-lowering effect due to an extensive enterohepatic recirculation to amplify the inhibition of cholesterol absorption [44]. The response of cholesterol homeostasis–related genes in the liver, such as HMGCR, SREBP1, SREBP2, PCSK9, and LDLR in the *ApoE*-deficient mice, also supported the similarity, since change of the 5 genes in response to high fat diet were all reversed after treatment with FANC and ezetimibe, respectively. Regarding the effects of oral route or intraperitoneal route of FANC administration in the hyperlipidemic mice, the results showed that, post FANC treatment, the decrement of total cholesterol and LDL-C, and the decrement of aortic plaque burden were comparable between the two routes, while the standard deviation of oral treatment group was wider, compared to intraperitoneal treatment group, suggesting a stable, continuous delivery of FANC into the animals by the osmotic minipumps rather than a discontinuous, intermittent delivery by oral intake of FANC. The only difference was lipid reduction in the liver, in which the intraperitoneal administration was more effective compared to oral route. Considering the daily intake of water is around 2 ml for each mouse, the daily dose of FANC given via oral route was about 4.5 fold of the intraperitoneal route.

### 4.2 FANC potentiate the action of DHLA against atherosclerosis

On the other hand, AuNPs of FANC did potentiate the action of DHLA, the reduced form of α-lipoic acid, in several aspects. Firstly, α-lipoic acid, has been shown to enhance ABCA1 expression and cholesterol efflux in mouse macrophages at concentrations more than 10 μM [45], 100 folds higher than the concentration of FANC used in the present study, to show the same effects in human macrophages. Secondly, in human umbilical vein endothelial cells, H_2_O_2_-induced expression of pro-inflammatory molecules ICAM-1 and VCAM-1 were reported to be suppressed by α-lipoic acid at concentrations of 100 μM or more [46], 1000 folds higher compared to FANC in the present study to show the comparable effects. Apart from cell culture experiments, in *ApoE*-deficient mice fed high fat diet, the aortic atheroma burden was reported to decrease nearly half by supplemented α-lipoic acid (0.2% wt/wt) in the diet, the reduced aortic atheroma burden was comparable to the present study with 300 nM FANC in the drinking water. Actually, in the safety assessment, oral administration of much higher dose of FANC did not show any form of morbidity or mortality at acute and subacute toxicity in both male and female ICR mice. The no observed adverse effect level (NOAEL) is considered 20 μM/100 μL/25 g mice [47]. Other evidences from the present study and our previous work [10] also showed that FANC attenuated markers of oxidative stress, such as MDA [48,49] and inflammatory cytokines, for example, TNFα, at a dose much lower than the comparable effects of α-lipoic acid reported by other investigators [50,51].

### 4.3 Anti-atherosclerotic effects of FANC beyond cholesterol-lowering

The findings that FANC markedly attenuated aortic atherosclerosis and mildly lowered serum cholesterol both in *ApoE-*deficient mice and *LDLr-*deficient mice supported that the properties of FANC beyond inhibition of cholesterol absorption in the intestine played a major role in the anti-atherosclerotic effects of FANC. Actually the present study unequivocally showed that FANC was able to decrease lipid burden in foam cells by inducing ABCA1 expression to promote reverse cholesterol transport [45]. Considering that FANC attenuated ox-LDL-induced macrophage attachment onto endothelial cells, and that FANC activated KEAP1-NRF2 system to turn on the expression of HO-1 to suppress the expression of ICAM-1 and VCAM-1, which are pro-inflammatory molecules of endothelium induced by ox-LDL present in hypercholesterolemia, FANC may play the very first step on inhibiting ox-LDL-induced ICAM-1 and VCAM-1 expression in endothelium *in vivo* to achieve the anti-atherosclerotic effects.

### 4.4 Mechanisms underlying the anti-inflammatory action of FANC at molecular level

The anti-inflammatory effects of FANC were explored according to their composition in the present study. Owing to DHLA is the capped ligands for the synthesis of colloidal AuNPs and DHLA was reported to bind the IRβ of insulin receptor [36], we tested and confirmed the binding of FANC to insulin receptor via the capped DHLA. In functional assay, similar to DHLA, FANC induced AKT phosphorylation, which was attenuated by IRβ depletion, indicating that FANC activated IRβ/AKT axis. Insulin was reported to exert an anti-inflammatory effect on HAECs via the suppression of ICAM-1 expression [52] through regulation of HO-1(39). Although insulin-induced HO-1 expression was AKT dependent, the present study showed that FANC induced-HO-1 expression was resistant to AKT inhibition, indicating an alternative activating node to induce HO-1 expression. Previous studies have shown that HO-1 suppressed vascular ICAM-1/VCAM-1 expression via NRF2 induction [53,54] and AuNPs induced HO-1 expression through NRF2 activation [55]. Therefore, the role of FANC on KEAP-NRF2 system was investigated in the present study. The results suggested that FANC bound KEAP1 via capped DHLA to promote KEAP1 degradation. As NRF2 is constitutively expressed, the decrease of KEAP1 would make NRF2 available for nuclear entry to initiate the expression of target gene HO-1 [19].

In conclusion, we found a novel cholesterol-lowering action of FANC in animal models of hypercholesterolemia and atherosclerosis. Multiple actions and mechanisms at cellular and molecular levels contributed to the anti-atherosclerotic effects, including inhibition of intestinal cholesterol absorption via interference with NPC1L1, enhanced cholesterol efflux in macrophages via ABCA1 up-regulation, and reduced attachment of macrophages onto endothelial cells by down-regulation of ICAM-1 and VCAM-1 through NRF2-HO-1 activation via KEAP1 degradation. Such cellular properties of FANC existed at dose of nano moles. On the other hand, the anti-atherosclerotic actions of FANC may involve other mechanisms and pathways and require further investigation. Nevertheless, all the findings, together with more available data regarding long-term safety of FANC, are worth testing FANC for clinical application against atherosclerosis.

## CRediT authorship contribution statement

**Yi-Nan Lee:** Writing – original draft, Data curation, Conceptualization. **Yih-Jer Wu:** Resources, Formal analysis. **Cheng-Huang Su:** Resources, Funding acquisition. **Bo-Jeng Wang:** Investigation, Data curation. **Sheng-Hsun Yang:** Funding acquisition, Data curation. **Hsin-I Lee:** Investigation, Data curation. **Yen-Hung Chou:** Investigation, Data curation. **Ting-Yi Tien:** Software, Resources, Methodology. **Chao-Feng Lin:** Visualization, Resources, Funding acquisition. **Wen-Hsiung Chan:** Investigation, Data curation, Conceptualization. **Ching-Hu Chung:** Resources, Investigation, Conceptualization. **Shin-Wei Wang:** Resources, Investigation, Conceptualization. **Hung-I Yeh:** Writing – review & editing, Supervision, Conceptualization.

## Funding

This work was supported by grants from 10.13039/100020595National Science and Technology Council of Taiwan (111-2314-B-195-024), and 10.13039/501100004861Mackay Memorial Hospital (MMH-E−111-03).

## Declaration of competing interest

The authors declare that they have no known competing financial interests or personal relationships that could have appeared to influence the work reported in this paper.

## Data Availability

No data was used for the research described in the article.
