# Supplementary material for: Fluorescent gold nanoclusters possess multiple actions against atherosclerosis
Source: Redox Biol. 2024 Nov 12;78:103427. doi: 10.1016/j.redox.2024.103427 (PMC11612375; doi:10.1016/j.redox.2024.103427)
Supplement: Multimedia component 1 [file mmc1.docx]

**Fluorescent gold nanoclusters possess multiple actions against atherosclerosis**

Yi-Nan Lee^1^, Yih-Jer Wu^1,2^, Cheng-Huang Su^1^, Bo-Jeng Wang^1^, Sheng-Hsun Yang^1^, Hsin-I Lee^2^, Yen-Hung Chou^2^, Ting-Yi Tien^1^, Chao-Feng Lin^1,2^, Wen-Hsiung Chan^3^, Ching-Hu Chung^2^, Shin-Wei Wang^4^, Hung-I Yeh^1,⁋^

^1^Cardiovascular Center, Department of Internal Medicine, and Department of Medical Research, MacKay Memorial Hospital, Taipei 10449, Taiwan.

^2^Department of Medicine, MacKay Medical College, New Taipei City 25245, Taiwan.

^3^Department of Bioscience Technology and Center for Nanotechnology, Chung Yuan Christian University, Zhongbei Road, Zhongli District, Taoyuan City 32023, Taiwan.

^4^Institute of Biomedical Sciences, MacKay Medical College, New Taipei City, Taiwan

**Supplement 1**


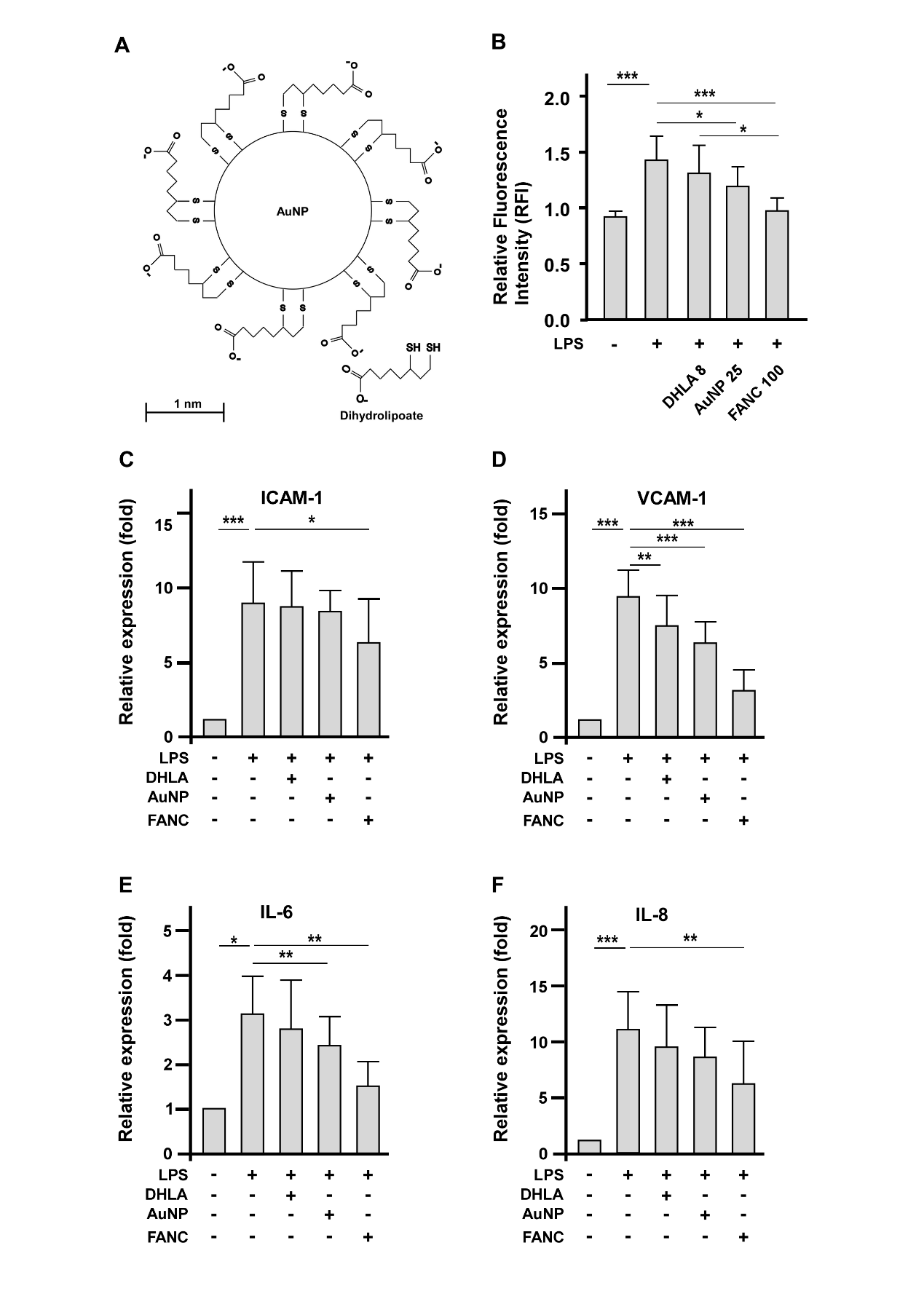


**Fig. S1 Comparisons of the antioxidant and anti-inflammatory activities among DHLA, AuNP and FANC.** (A) schematic diagram of FANC structure. The number of capped DHLA surrounding a gold particle is about 80 but shows 8 for simplification (Refer to Supplementary Method for the component gold and DHLA in FANC). Scale bar, 1 nm. (B) Comparison of the antioxidant activities among DHLA, AuNP (1.9 nm, catalog #1102; Nanoprobes, Inc, NY) and FANC. HAECs were pretreated with indicated DHLA (μM), AuNP (μM) and FANC (nM) for 72 h, followed by 100 ng/ml of LPS treatment overnight. Intracellular reactive oxygen species (ROS) was measured using the cell permeant reagent 2’,7’-dichlorofluorescin diacetate (DCFDA) to quantitatively assess ROS in live cells (oxidative stress assay kit, ab113851, abcam). (C) to (F), Comparisons of the anti-inflammatory activities among DHLA (8 μM), AuNP (25 μM) and FANC (100 nM). HAECs were pretreated with indicated DHLA, AuNP and FANC for 72 h, followed by 100 ng/ml of LPS treatment overnight. Relative expression of ICAM-1, VCAM-1, IL-6 and IL-8 were measured by quantitative PCR (qPCR). Values are mean ± SD of triplicate assays from 3 independent experiments. *p<0.05; **p<0.01; ***p<0.001

**Supplement 2**

**
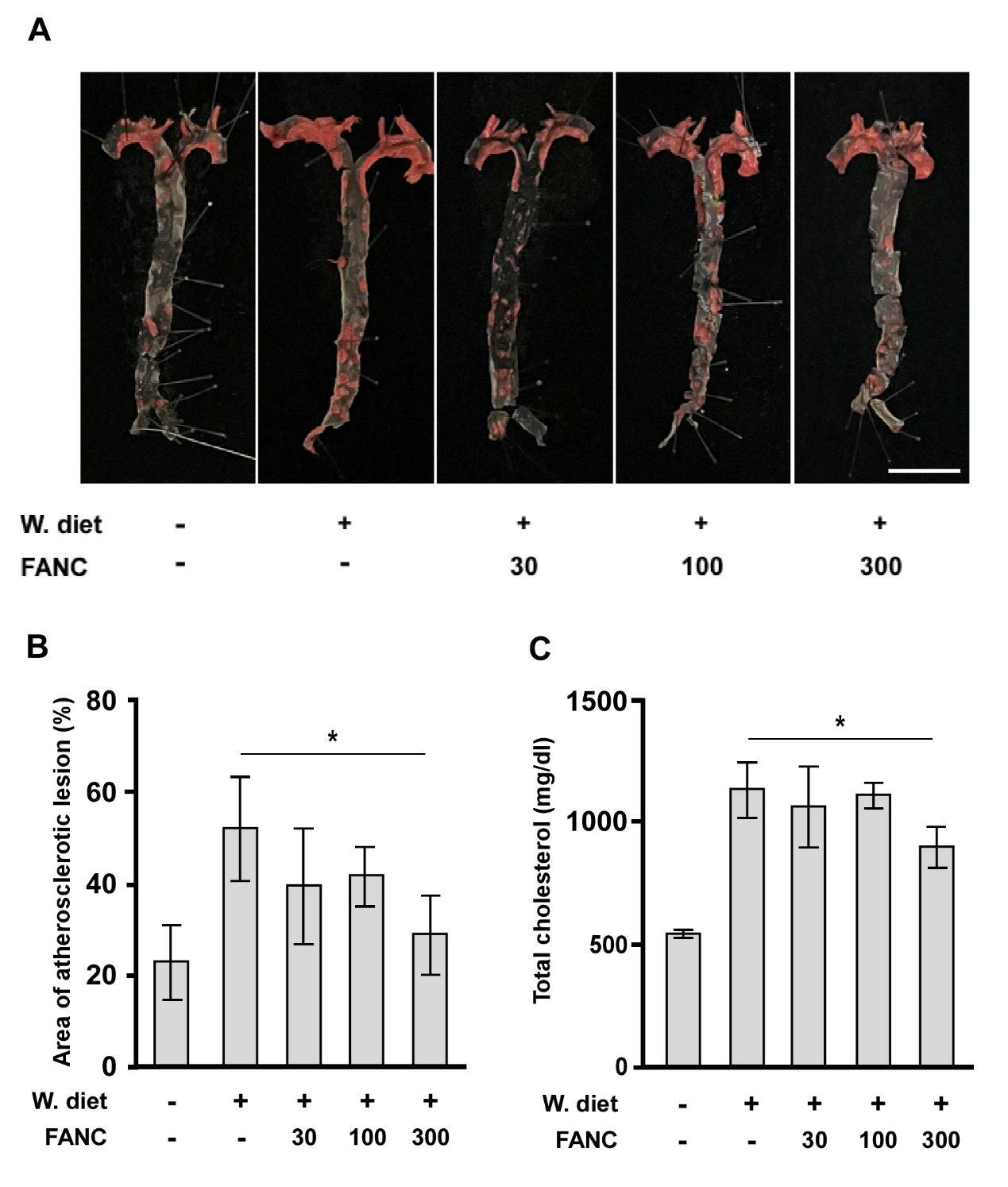
**

**Fig. S2 Dosage tests of oral FANC administration on Western-type diet-induced atherosclerotic lesions and hypercholesterolemia in *ApoE*-deficient mice**

Eight-week old *ApoE*-deficient mice were fed with Western-type diet (W. diet) for 8 weeks. FANC were concomitantly supplemented in drinking water at indicated concentrations (nM) for 8 weeks. (A) Arterial trees were stained with Sudan IV (red) to detect atherosclerotic lesions. n=3 for each group. Scale bar, 0.5 cm. (B) Quantification of atherosclerotic lesion area in arterial trees. (C) Effects of FANC in drinking water on W. diet-induced hypercholesterolemia. Sera were collected at the end of experiment. *, p<0.05.

**Supplement 3**


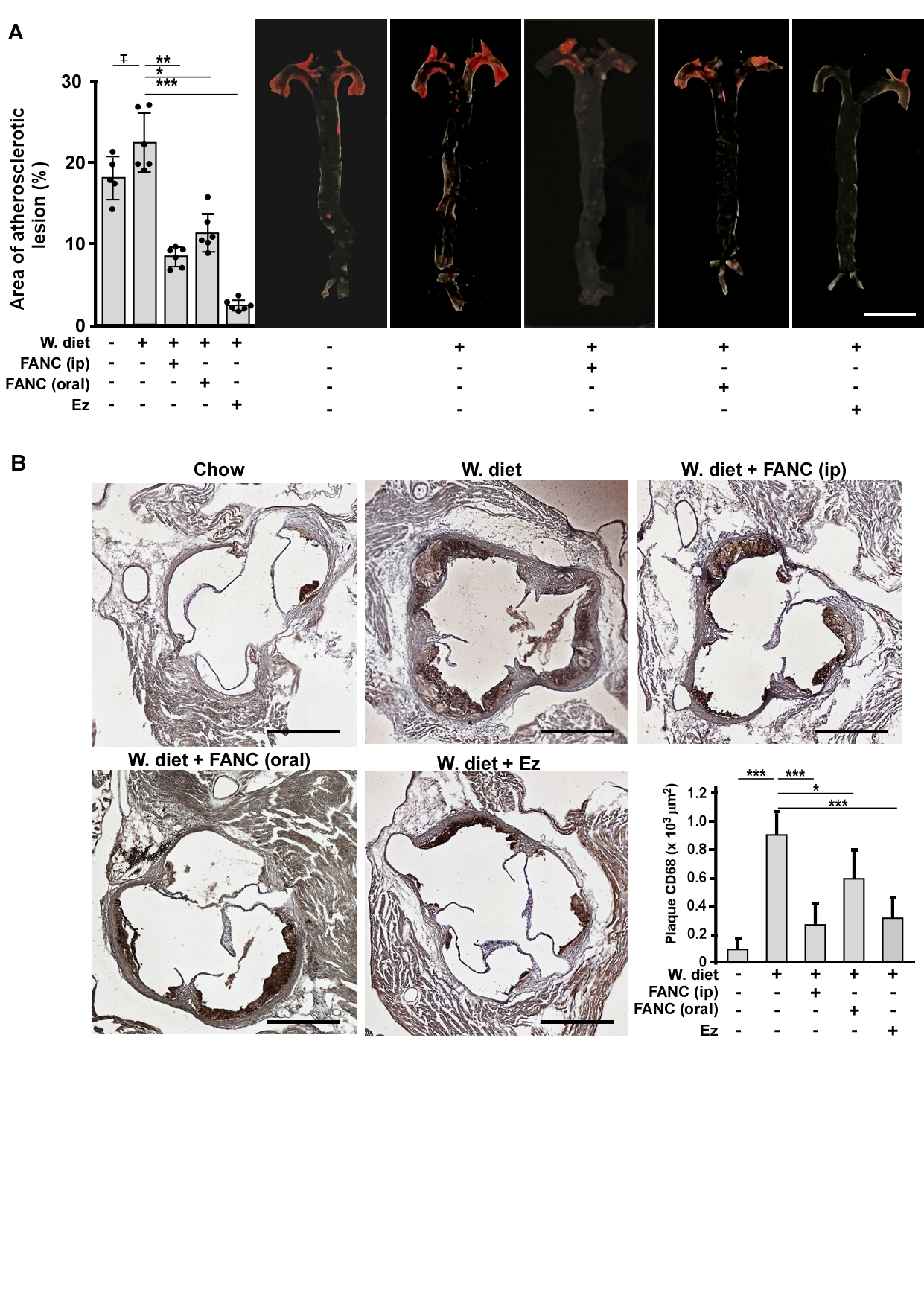


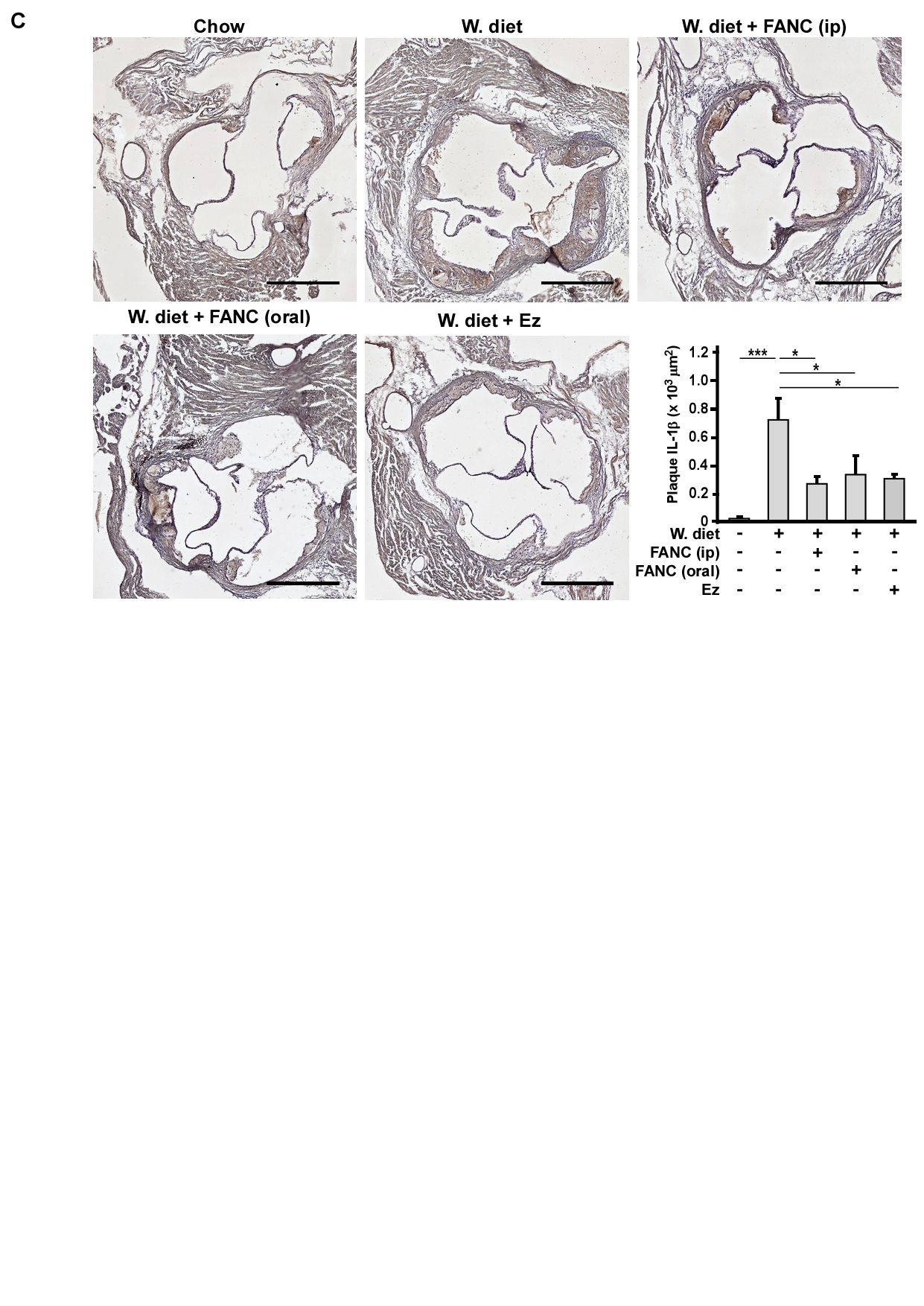


**Fig. S3 Effects of different FANC dosage and administration routes on Western-type diet-induced atherosclerotic lesions and pro-inflammatory responses in *ApoE-*deficient mice**

Animals were fed with Western-type diet (W. diet) and treated with FANC intraperitoneally (FANC (ip)) or orally (FANC (oral, 300 nM) for 8 weeks. Ezetimibe (Ez) was used as control. (A) Arterial trees were stained with Sudan IV (red) and quantified to detect atherosclerotic lesions. n=5 for chow-fed mice and n=6 for W. diet-fed animals. Scale bar, 0.5 cm. Aortic sinuses were sectioned and stained with CD68 (B) and IL1-β (C). The signals appearing in plaque areas were measured and quantified. n=3 in each group. Scale bar, 500 μm. Values are mean ± SD of triplicate assays from 3 independent experiments. *p<0.05; ***p<0.001

**Supplement 4**


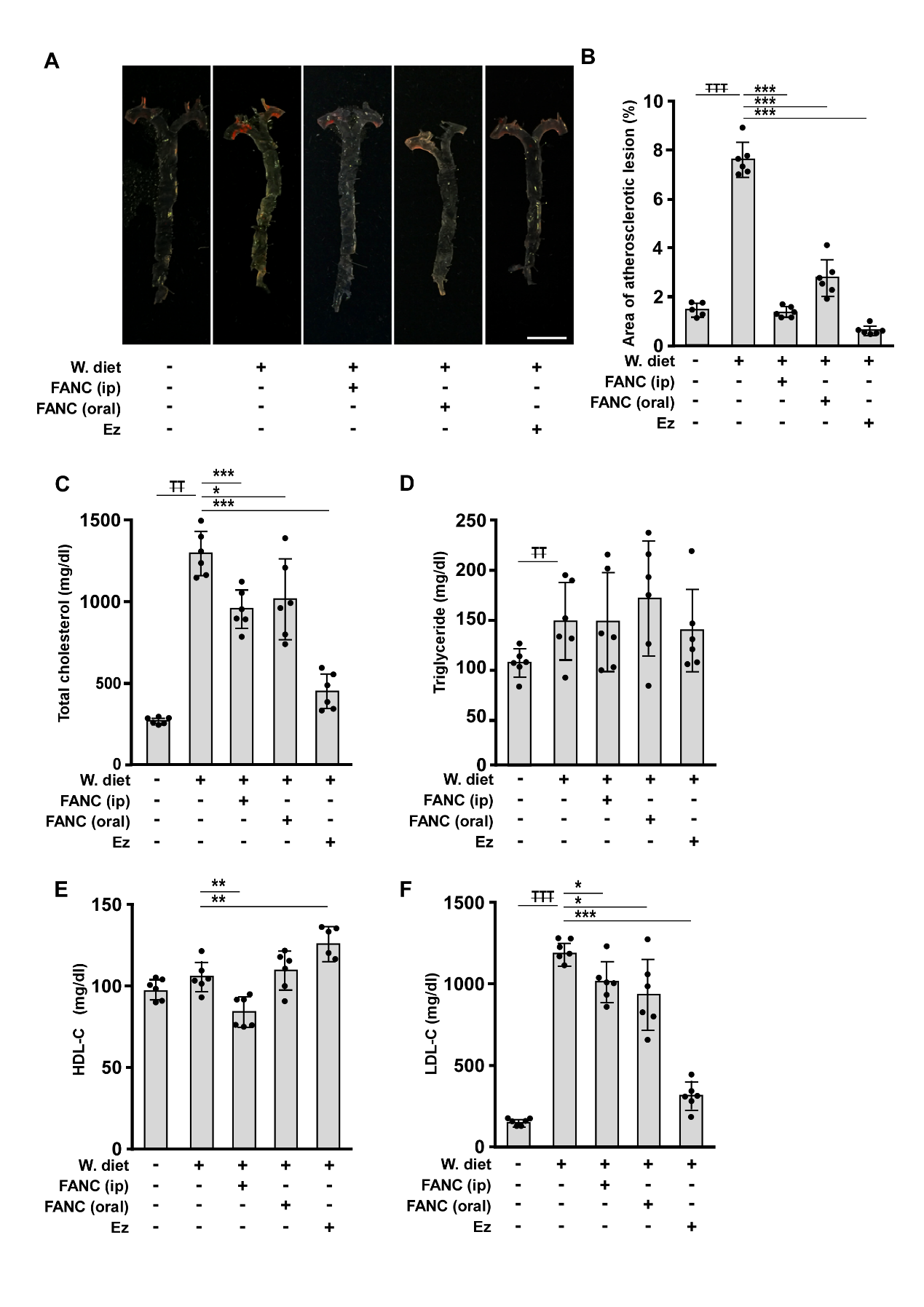
**Fig. S4 Comparison of FANC administration routes on Western-type diet-induced atherosclerotic lesions and lipid profile changes in *LDLR-*deficient mice**

Eight-week old *LDLR*-deficient (*LDLR^-/-^*) mice were fed Western-type diet (W. diet) for additional 8 weeks. FANC were concomitantly given by intraperitoneally implanted osmotic minipumps (FANC (ip)) or by drinking water (FANC (oral) containing 300 nM FANC for 8 weeks. Ezetimibe (Ez, 0.005% w/w) was supplemented with W. diet for 8 weeks. (A) Arterial trees were stained with Sudan IV (red) to detect atherosclerotic lesions. n=6 for each group. Scale bar, 0.5 cm (B) Quantification of atherosclerotic lesion area in arterial trees. (C) to (F) Lipid profiles of different FANC administration routes in *LDLR-*deficient mice. Sera were collected at the end of experiment to measure total cholesterol, triglycerides, HDL-C and LDL-C. ^~~T~~^, compared with chow group; *, compared with W. diet group. ^~~T~~^ and *, p<0.05; ^~~TT~~^ and **, p<0.01; ^~~TTT~~^ and ***, p<0.001.

**Supplement 5**

**
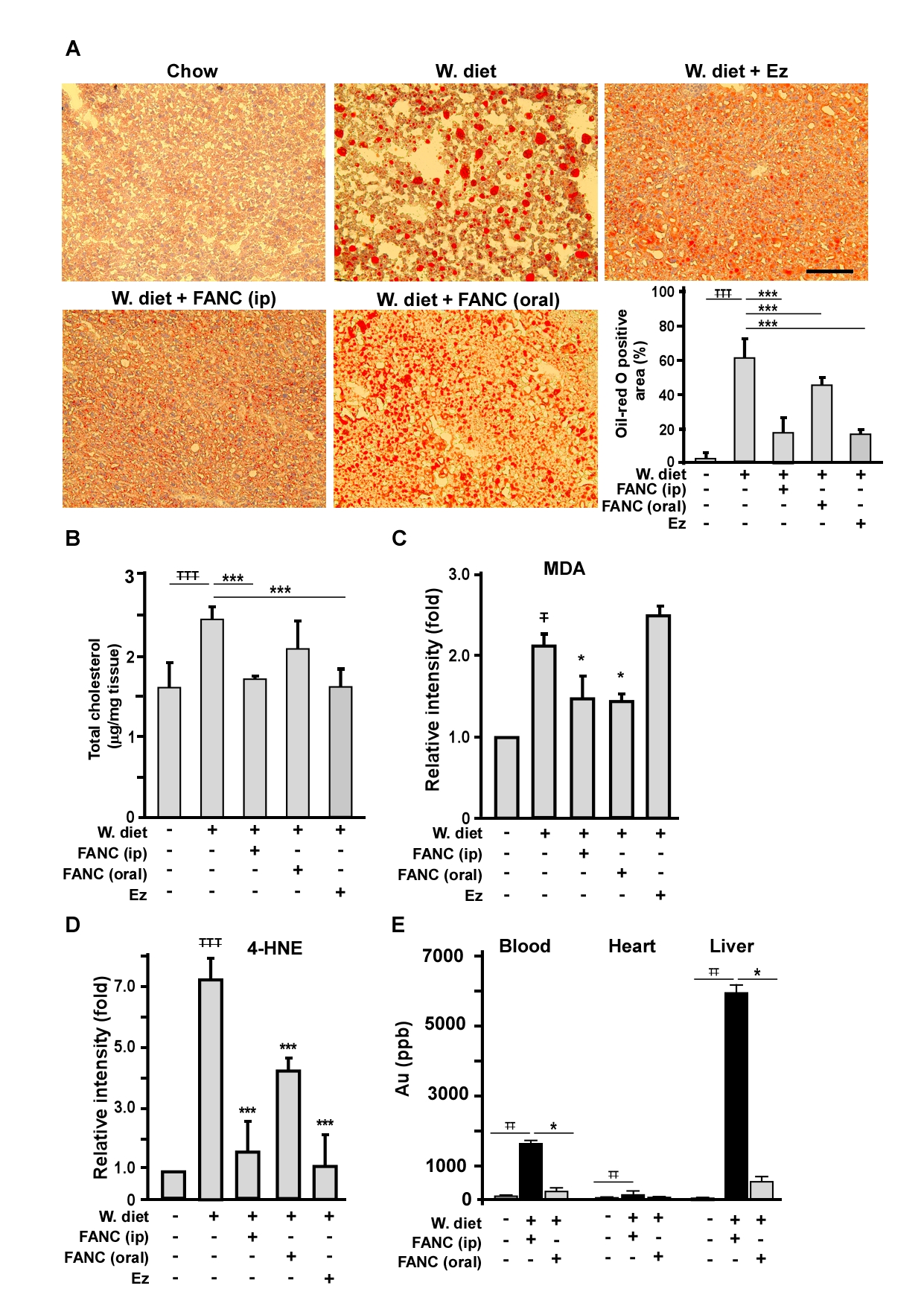
**

**Fig. S5 Effects of FANC on hepatic lipid accumulation and serum lipid peroxidation, and the organ and tissue levels in *ApoE-*deficient mice fed Western diet**

(A) Liver sections of *ApoE^-/-^* mice with indicated treatments were stained with Oil-red O and quantified to determine lipid deposit, n=3. Scale bar, 20 μm. (B) Total cholesterol extracted from liver. n=3. ^~~T~~^, compared with vehicle; *, compared with W. diet group; ^~~TTT~~^ and ***, p<0.0 01. (C) Serum MDA and (D) 4-HNE level. (E) Inductively coupled plasma-mass spectrometer (ICP-MS) determination of gold nanoparticle level in blood, heart and liver of animals with or without FANC administration. Detection limit, 0.6 ppb. n=3. ^~~T~~^, compared with chow group; *, compared with W. diet group. ^~~T~~^ and *, p<0.05; ^~~TT~~^ and **, p<0.01; ^~~TTT~~^ and ***, p<0.001

**Supplementary Methods:**

**Composition of gold and DHLA in FANC**

Molecule weight (MW) of Au is 197 g/mole and DHLA is 206.33 g/mole.

Weight of 1 M of FANC per liter is ranged from 47100 g to 65700 g. Accordingly, 1 M of FANC are esteemed containing 200 to **250** moles of Au,

and 37 to **80** moles of DHLA.

Converting table of gold and DHLA in FANC

| **FANC** | **Au** | **DHLA** |
| --- | --- | --- |
| **1 M** | **250 M** | **80 M** |
| **100 nM** | **25 μM** | **8 μM** |

**Quantitative reverse transcription-PCR**

HAECs were pretreated with indicated DHLA, AuNP and FANC for 72 h and followed with of LPS (100 ng/ml) overnight. Total RNA was harvested using the RNeasy Plus mini kit (Qiagen, Germany). The first strand cDNA was synthesized from 1 μg of total RNA using the SuperScript III First-Strand Synthesis System kit (Invitrogen, USA). Real-time PCR were amplified with primers specific for ICAM-1, VCAM-1, IL-6 and IL-8 using iQ SYBER Green Supermix reagent and detected with the iQ single-color real-time PCR detector system (all from Bio-Rad, USA). Data were analyzed with iQ5 optical system software, Version 2.0 (Bio-Rad). Relative mRNA levels were normalized with the corresponding levels of β-actin.

ICAM-1 sense strand primer: 5’-CTGACCCCAACCCTTGATGAT-3’

ICAM-1 antisense strand primer: 5’-AGCCCCATTTGATCTTTTTGC-3’

VCAM-1 sense strand primer: 5’-CGGATTGCTGCTCAGATTGGAGAC-3’

VCAM-1 antisense strand primer: 5’-AAACTCACAGGGCTCAGGGTCAG-3’

IL-6 sense strand primer: 5’-GCTTTAAGGAGTTCCTGC-3’

IL-6 antisense strand primer: 5’-GGTAAGCCTACACTTTCCA-3’

IL-8 sense strand primer: 5’-CCACTGTGCCTTGGTTTC-3’

IL-8 antisense strand primer: 5’-TCTTGCACAAATATTTGATGC-3’

β-actin sense strand primer: 5’-CCTCCCTGGAGAAGAGCTACGA-3’

β-actin antisense strand primer: 5’-CGCCAGACAGCACTGTGTTG-3’

**Immunohistochemistry**

OCT-embedded aortic sinuses were sectioned at 10 μm and left air dry for 1 hr. Tissue slides were fixed with 4% paraformaldehyde (PFA) for 10 min and followed with the times of PBS washes. For Oil-red O (ORO) staining, tissues slides were incubated with 60% isopropanol for 5 min. ORO working solution was prepared from stock (0.5% in isopropanol) diluted with ddH_2_O in 3:2 ratio. ORO working solution was filtered by 0.22 μm discs. Tissue slides were stained with 0.22 μm filtered ORO working solution for 15 min. ORO stained slides were washed with running tap water for 10 min and counterstained with hematoxylin.

For CD68 and IL-1β staining, PFA fixed slides were blocked with 10% horse serum for 1 hr and incubated with primary antibodies (1 : 100 dilution) overnight. After 0.2% Tween-100 in PBS wash for 5 min, tissue slides were incubated with corresponding HRP-conjugated secondary antibodies for 1 h. After 5 min of 0.2% Tween-100 wash, images were development by Liquid DAB + Substrate Chromogen system (# K3468, DAKO).

**Hepatic** **lipid extraction** **and determination**

Fresh harvested liver tissues were diced, weighted and snapped frozen by liquid nitrogen. Lipid extraction was performed using the method described by Hara and Radin [1] with certain modification [2]. Fifty mg of tissue was introduced into a glass tube with a spiral stopple and then mixed with 1 ml of hexane: 2-propanol (3;2, v/v). The tubes were filled with nitrogen gas to minimize lipid oxidation before being closed. The tubes were then left for orbital agitation overnight under dark. The extraction was filtered wit 0.22 μm disc to remove tissue remnants. Filtered extraction was added 0.3 ml of Na_2_SO_4_ to make 0.47 M final concentration. Tubes were vortex for 5 min, left for 15 min in orbital agitation and centrifuged at 1000g for 10 min at 4°C. The upper phase of each tube was transferred to a pre-weighted glass tube and left dried with nitrogen gas. The dry extracts were dissolved in 50 μl of LPL buffer (28.75 mM Pipes; 57.41 mM MgCl_2_^.^6H_2_O) with 0.1% SDS. Lipid measurement from dry extract was determined by enzymatic methods using Cholesterol Quantificatin Assay kit (CS0005, Sigma-Aldrich). 2 μl of lipid extract was added to 48 μl of Colorimetric Reaction Mix and incubated at 37°C for 30 min. Measure the absorbance at 570 nm (A570).

**Serum MDA and 4-HNE determination**

MDA was determined by using lipid peroxidation (MDA) assay kit (abcam, ab233471). Fifty μl of test serum and serially diluted MDA standards were added into a 96-well clear bottom microplate. Add 10 μL MDA Color Reagent solution into each well of MDA standard, blank control, and test serum. Incubate the reaction mixture at room temperature for 30 minutes. Add 40 μL of Reaction Solution to each well and incubate at room temperature for 1 hr. Measure end-point absorbance at OD 695 nm.

4-HNE by using lipid peroxidation (4-HNE) ELISA kit (abcam, ab238538). Add 50 μL standard or serum to 4-HNE conjugated 96-well plate and incubate for 10 mins. Add 50 μL of the diluted anti-4-HNE antibody and incubate for 1 h. After wash with 200 μL of Wash Buffer, add 100 μL of HRP conjugated secondary antibody and incubate for 1 h. After wash with 200 μL of Wash Buffer, add 100 μL of Substrate Solution and incubate for 20 mins. Add 100 μL of Stop Solution and read absorbance at 450 nm.

**Tissue gold nanoparticle determination**

Tissues (bloods, hearts and livers) were harvested, weighted and kept at -20°C. Samples were sent to Instrumentation Center at National Tsing Hua University for gold level determination. In brief, Samples were warmed to room temperature for approximately 2 h, then add 8 mL HNO_3_ and 2 mL HCl into each microwave vessel using a microwave digestion system (CEM MARS 6 iWave, Matthews, NC). Sample analysis was performed using an inductively coupled plasma-mass spectrometer (Thermo Fisher Scientific iCAP TQ). Every sample was measured in triplicate. DELL OptiPlex XE3 Workstation was used for data analysis

**Supplementary References:**

1. Hara, A. and N.S. Radin, *Lipid extraction of tissues with a low-toxicity solvent.* Anal Biochem, 1978. **90**(1): p. 420-6.

2. Rodríguez-Sureda, V. and J. Peinado-Onsurbe, *A procedure for measuring triacylglyceride and cholesterol content using a small amount of tissue.* Anal Biochem, 2005. **343**(2): p. 277-82.
